# Companion: A Pilot Randomized Clinical Trial to Test an Integrated Two-Way Communication and Near-Real-Time Sensing System for Detecting and Modifying Daily Inactivity among Adults >60 Years—Design and Protocol

**DOI:** 10.3390/s23042221

**Published:** 2023-02-16

**Authors:** Diego Arguello, Ethan Rogers, Grant H. Denmark, James Lena, Troy Goodro, Quinn Anderson-Song, Gregory Cloutier, Charles H. Hillman, Arthur F. Kramer, Carmen Castaneda-Sceppa, Dinesh John

**Affiliations:** 1 Bouvé College of Health Sciences, Northeastern University, Boston, MA 02115, USA; 2 Philadelphia College of Osteopathic Medicine, Philadelphia, PA 19131, USA; 3 Manning College of Nursing and Health Sciences, University of Massachusetts Boston, Boston, MA 02125, USA; 4 College of Science, Northeastern University, Boston, MA 02115, USA; 5 Beckman Institute, University of Illinois, Urbana, IL 61801, USA

**Keywords:** sedentary behavior, physical inactivity, move more and sit less, older adults, near-real-time interventions, sensor-based physical activity measurement, mHealth interventions, pilot randomized controlled trials, study protocols

## Abstract

Supervised personal training is most effective in improving the health effects of exercise in older adults. Yet, low frequency (60 min, 1–3 sessions/week) of trainer contact limits influence on behavior change outside sessions. Strategies to extend the effect of trainer contact outside of supervision and that integrate meaningful and intelligent two-way communication to provide complex and interactive problem solving may motivate older adults to “move more and sit less” and sustain positive behaviors to further improve health. This paper describes the experimental protocol of a 16-week pilot RCT (N = 46) that tests the impact of supplementing supervised exercise (i.e., control) with a technology-based behavior-aware text-based virtual “Companion” that integrates a human-in-the-loop approach with wirelessly transmitted sensor-based activity measurement to deliver behavior change strategies using socially engaging, contextually salient, and tailored text message conversations in near-real-time. Primary outcomes are total-daily and patterns of habitual physical behaviors after 16 and 24 weeks. Exploratory analyses aim to understand Companion’s longitudinal behavior effects, its user engagement and relationship to behavior, and changes in cardiometabolic and cognitive outcomes. Our findings may allow the development of a more scalable hybrid AI Companion to impact the ever-growing public health epidemic of sedentariness contributing to poor health outcomes, reduced quality of life, and early death.

## 1. Introduction

Supervised exercise with a trained instructor [1,2] is most effective in maximizing health benefits, is popular among older adults >60 years, and is most common in physical activity interventions aimed at preventing aging-related disability and disease [3,4,5,6,7,8]. Supervision results in higher training effects and program adherence [5,6,9,10] due to personal attention and interaction with the trainer that motivates participants [6,9]. Drawbacks of the model include a low frequency of trainer contact (60 min, ~1 to 3 times/week) and ineffectiveness in changing behavior outside weekly sessions [6]. Additionally, older adults may compensate for increased exercise by increasing sedentary behavior outside training sessions, which attenuates positive gains in health (e.g., improved energy metabolism, musculoskeletal, functional, cardiovascular, and psychosocial health, etc.) from supervised exercise [8,11,12,13,14,15,16,17]. Independent of physical activity, reducing sedentary time improves cardiometabolic and functional health in older adults [18,19] and correspondingly, federal guidelines now recommend that older adults “move more and sit less” during the day [20]. Thus, strategies to motivate older adults who participate in supervised programs to be more active and sit less outside structured weekly training sessions are necessary. However, there are limitations to increasing physical activity among older adults (e.g., difficulties in accessing health facilities and professionals).

Technologies including wearables and smartphones have the potential to promote daily positive behaviors in older adults [21]. While several interventions incorporate these technologies to modify behavior, there is limited work on the integration of these technologies to generate meaningful two-way communication [22] that may enable an interventionist to successfully engage an older adult through timely, intelligent, and context-sensitive dialogue to problem solve and motivate behavior change. Awareness of ongoing or recent behavior (from wearable sensors), knowledge of the contexts of behavior/past conversations, and individual’s preferences (gathered via two-way human-to-human communication) may help in generating meaningful discourse between the interventionist and an older adult that allows the creation of an adaptive intervention at a level that is not currently possible. We have integrated wearable sensors with the smartphone to develop a behavior-aware, virtual “Companion” that uses a human-in-the-loop [23] approach to enable meaningful two-way communication. Such an intensive longitudinal data gathering approach during a behavioral intervention may enable the building of rich models of typical behavior to deliver intervention components and behavior change strategies using socially engaging, contextually salient, and tailored text message conversations in near-real-time.

The purpose of this paper is to describe the experimental protocol to pilot test the efficacy of the Companion in motivating older adults to “move more and sit less”. We will deploy Companion in a randomized clinical trial (N = 46) [24] (Stage I of NIH Stage Model) [25,26] for 16 weeks with follow-up after 24 weeks to test if it improves and sustains free-living physical activity and sedentary behaviors in older adults. Primary endpoints are total-daily and patterns of habitual physical activity and sedentary behaviors measured at baseline and after the intervention. Exploratory endpoints include continuously measured (16-week) physical behavior outcomes, Companion’s user engagement and its relationship with continuously measured physical behaviors, and cardiometabolic health, body composition, cognition, and psychosocial outcomes measured at baseline and after the intervention.

## 2. Materials and Methods

### 2.1. Hypotheses and Experimental Design

Our long-term goal is to develop a semi-automated hybrid artificial intelligence (AI) system with a human-in-the-loop approach for delivering adaptive interventions to reduce the burden of chronic disease resulting from sedentary behaviors and physical inactivity in older adults. Pilot testing the efficacy of the current Companion will provide proof of concept for a subsequent Stage II NIH Stage Model [25,26] study that will aim to establish the efficacy and scalability of a hybrid AI-based Companion among older adults.

In the current study, Companion is a meta-intervention deployed along with a supervised exercise program and will be experienced by the user as their “intelligent” human trainer providing extended behavior change support outside face-to-face personal training sessions. Companion will enable the building of contextualized and continuously refined visualizable models of typical behavior derived using machine learning-based processing of passive wearable sensor data and interactive two-way communications. These models will be used to determine appropriate opportunities to deliver intervention components and behavior change strategies using socially engaging, contextually salient, and tailored text message conversations in near-real-time.

This is a small sample (N = 46) 16-week parallel randomized clinical trial with follow-up after 24 weeks in sedentary older adults. Participants will be evenly randomized into a treatment or control group. All participants will engage in a supervised exercise program (2 times/week) over the 16-week study period that has been proven to help prevent aging-related disability and disease [27,28]. Only the treatment group (N = 23) will receive Companion from weeks 1 to 16. The study will be carried out by researchers at Northeastern University’s Human Performance and Exercise Science Lab in Boston, MA, and participants will be recruited from the Greater Boston metro area. The study is registered on ClinicalTrials.gov (Identifier: NCT04827212). Ethics approval was granted by Northeastern University’s Institutional Review Board.

The primary aim will assess the *a priori* hypothesis that the treatment group will show greater improvements in daily habitual activity and sedentary behavior outcomes than controls after 16 and 24 weeks. The exploratory aims are to (i) assess the exploratory hypothesis that the treatment group will show greater improvements in continuously measured (16 weeks) daily physical behavior outcomes, (ii) conduct an exploratory analysis of Companion’s user engagement and its relationship to continuously measured daily physical behaviors, and (iii) assess the exploratory hypothesis that the treatment group will show greater improvements in health outcomes after 16 and 24 weeks.

### 2.2. Rationale and Feasibility Testing

The approach to this study is guided by evidence from prior relevant work [1,5,6,8,9,10,11,12,13,14,15,16,17,27,28,29] and preliminary feasibility testing of the Companion concept in our lab.

#### 2.2.1. Supervised Exercise Programs Fail to Increase Daily Habitual Behavior

Supervised exercise programs are the most effective means of maximizing adherence and training effects [5,6,9,10] of sedentary older adults because human contact in such programs supports known facilitators of exercise engagement [1,29] in older adults (e.g., social interaction, increased awareness of the psychosocial and physical benefits of becoming more active, professional one-to-one instruction, a collaboratively agreed program design, and increased confidence to partake in physical activity). However, a low frequency of trainer contact (60 min, ~1 to 3 times/week) due to a high resource intensiveness of such programs coupled with evidence that older adults may compensate for increased exercise by increasing sedentary behavior outside training sessions [8,11,12,13,14,15,16,17] means that such programs may do little to reduce the burden of sedentariness in older adults. For example, (i) exercise referrals have a small absolute population-level risk reduction effect on sedentary behavior with approximately 1 in 17 referrals resulting in a moderately active lifestyle [8], and (ii) participation in exercise training results in no statistically or clinically significant increases on average in the non-exercise physical activity (i.e., activities of daily living) of older adults [17,30]. Older adults may fail to engage in physical activity outside of periods of supervision due to the same barriers [8] that result in an exercise program referral in the first place, e.g., lack of self-efficacy, lack of social support, feelings of intimidation with exercise and/or physical activity, poor time management, etc. Leveraging personal human contact and extending the frequency of trainer–trainee interactions from such programs is thus vital to motivate an increase in daily habitual physical activity.

#### 2.2.2. Feasibility Testing

We used two health coaches and six older adults (mean age ± SD: 67.7 ± 6.2 years) in a single-case experimental design study over three months to obtain an understanding of the ability of two-way communication in engaging participants and the contexts of communication [31]. Companion was successful in eliciting an 85% two-way interaction rate and the following communication contexts: 26% physical activity related, 38% health related, 19% social, 11% diet related, 5% behavior related, and 1% sleep related [31].

### 2.3. Participants

#### 2.3.1. Inclusion and Exclusion Criteria

Inclusion criteria are (i) age > 60 years, (ii) BMI > 25 kg/m^2^, (iii) no health conditions preventing participation in physical activity lasting 10 to 30 min, and (iv) having a smart phone. Screened individuals will be excluded from participation if any of the following conditions are met: (i) engagement in structured physical activity on more than two days/week lasting ≥ 30 min/session, (ii) regular use of an assistive device for walking, and (iii) if participation in an exercise program is likely to alter medications pertaining to cardiovascular or metabolic health.

#### 2.3.2. Recruitment, Screening Procedures and Randomization

**Figure 1:**
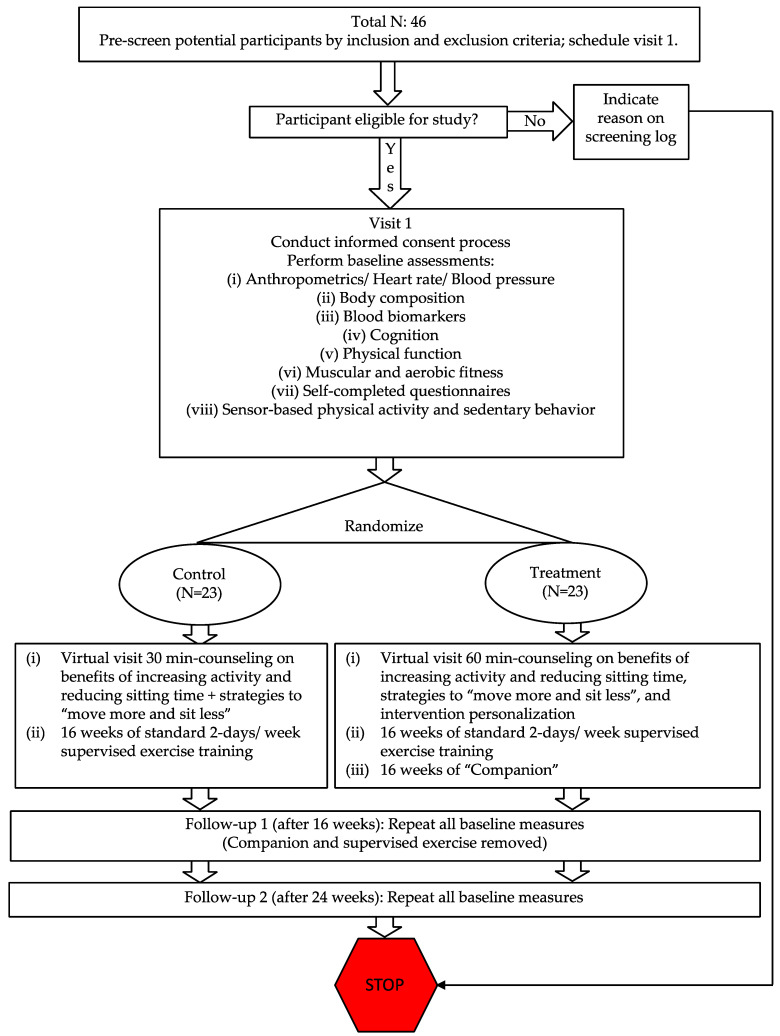
Study flow diagram.

Potential participants will be sought from the Greater Boston metro area using flyers, emails, and in-person plus telephone outreach to various surrounding organizations and communities, including eligible older adults who participated in prior studies at Northeastern University. Participants will begin baseline assessments upon provision of signed and dated written consent. Figure 1 contains the flow diagram for the study that outlines the phases of our RCT. Detailed recruitment and retention, screening, and consent procedures are in our study protocol that is provided in the Supplementary Materials.

Potential participants who meet eligibility criteria and who provide informed consent will be randomly assigned in a 1:1 ratio to either the control (N = 23) or treatment (N = 23) condition using a random coin flip scheme on Excel.

### 2.4. Measurements

Here, we briefly describe all outcome measurements that are assessed at baseline and after 16 and 24 weeks (see supplementary study protocol). Participants will be asked to fast for 12 h prior to conducting health assessments.

#### 2.4.1. Primary Outcomes

*Sensor-based physical activity and sedentary behavior*: Habitual total and bouts of physical activity (intensity and type) and sedentary behaviors will be measured using a continuously worn thigh sensor for one week at baseline and follow-up assessments. A week-long measurement is representative of habitual behavior [32,33]. For these measurements, (i) an activPAL^TM^ 3C activity monitor (PAL Technologies Ltd., Glasgow, UK) will be adhered to the dominant thigh with a hypoallergenic tape. Additionally, participants will be asked to complete a daily log to self-report time-in-bed (night-time sleep and daytime naps) and when the monitors are not worn (e.g., showering, swimming). Thigh sensor data will be processed using proprietary software (PALanalysis version 8, PAL Technologies Ltd., Glasgow, UK) and algorithms (i.e., CREA; PAL Processing Suite) [34,35,36,37,38,39] to obtain free-living habitual measures of physical activity and sedentary behavior.

#### 2.4.2. Exploratory Outcomes

Detailed measurement procedures for all exploratory outcomes are available in the supplemental study protocol. (i) *Continuously measured (16 weeks) physical activity, sedentary behavior, and sleep*: These outcomes will be measured by wrist-worn sensors of the Companion system (described in detail below) in the treatment group and using an ActiGraph GT9X (ActiGraph Corp, LLC, Pensacola, FL, USA) in the control group. These devices will be worn on the non-dominant wrist. Wrist sensor data will be processed using Monitor-Independent Movement Summary unit (MIMS) to yield overall movement volume estimations [40] and machine learning algorithms to yield sensor wear, sleep, and wake behavior characterization (i.e., sedentary, ambulation, and upright behaviors) [41,42,43,44] during the 16-week intervention period. (ii) *Health outcomes*: (a) anthropometrics (height, weight, and waist/hip circumference); (b) body composition (via bioelectrical impedance) [45]; (c) seated resting heart rate and blood pressure; (d) fasting blood biomarkers (blood glucose and lipid profile); (e) cognition (via The NIH Toolbox Cognition Battery) [46]; (f) physical function (via The Short Physical Performance Battery) [47,48]; (g) upper and lower body muscle endurance (via maximal push-up and squat tests, respectively) [49]; (h) aerobic fitness (via the six-minute walk test) [50,51]; and (i) other questionnaire-based health outcomes (see supplementary study protocol). These will be self-administered electronically following baseline and follow-up assessments and will consist of health history (baseline only), dietary intake [52,53], and psychosocial outcome measures (i.e., perceived competence scale for participating in regular physical activity [54,55], short health-care climate questionnaire [54], exercise self-regulation questionnaire [56,57], and basic needs satisfaction relationship scale [58]). Companion is based on self-determination theory (SDT) and measuring psychosocial outcomes will enable the assessment of how constructs of SDT [59] relate to participation in physical activity [60].

### 2.5. Control and Intervention Components

Both the control and treatment groups will receive virtual counseling prior to commencing the study and subsequent access to supervised exercise training.

#### 2.5.1. Virtual Counseling

Following baseline assessments, both groups will receive a ~30 min face-to-face virtual (i.e., Zoom Video Communications, Inc., San Jose, CA, USA) counseling session on the benefits of decreasing sedentary behavior and increasing physical activity and on strategies to “move more and sit less” [61,62,63]. In addition to this, the treatment group will participate in a second ~30 min interview to enable information gathering to support intervention personalization. This information will pertain to (i) physical activity, fitness, and/or health-related goals; (ii) barriers to engaging in regular physical activity and/or exercise; (iii) typical schedules/behavior routines during work, leisure, home, etc.; (iv) preferences related to physical activity and exercises; (v) hobbies and leisure time interests; (vi) work and/or home environments and available opportunities/resources in these environments to support regular participation in physical activity; (vii) family and household dynamics; and (viii) commuting needs and preferences.

#### 2.5.2. Supervised Exercise Training

The 16-week supervised exercise training plan that both groups will receive is based on guidelines from the American College of Sports Medicine (ACSM) [64,65]. Participants will participate in virtual Zoom-based structured exercise sessions (~45–60 min) led by an ACSM certified personal trainer and a trained exercise physiologist on two days/week that include cross-training activities to improve cardiorespiratory endurance, muscular strength and mass and endurance, flexibility, and balance. Supervised training sessions for the control and treatment groups will be scheduled separately to avoid contamination of study arms. We will use a virtual format due to the COVID-19 pandemic and to minimize the participant burden of coming to the lab.

Program variables (i.e., choice of resistance, exercise selection and sequence, number of sets and repetitions, frequency, and rest period length) will be manipulated following the principles of progressive overload resistance training [66]. A cautionary, monitored, and individualized approach to increasing training demands on the participants will be employed based on initial training status, past experience, joint health, and response to training [66]. Sample training routines at various stages of the progression model are available online (i.e., “Companion Study: Exercise Prescription Database”; https://www.youtube.com/channel/UCZsg8fPtquiVrsv8INIvC0g (accessed on 11 November 2022)) [67]). Table 1 outlines the program’s overall rate of progression [66]. Additional descriptions on the training program and its progression are available in the supplementary study protocol.

Additionally, control group participants will engage in goal setting with the trainer and receive exercise and physical activity prescriptions to be performed on their own after each training session [49,65].

#### 2.5.3. The Companion

Here, we describe the theoretical underpinnings of the Companion system, its technical components, and the integration of these to deliver tailored messages and interventional strategies.

*Theory*: Companion is based on an SDT framework via the application of motivational interviewing (MI) to creatively use information on behaviors, attitudes, and environmental contexts to create participant–Companion interactions that are tailored to the former’s needs. SDT addresses cognitive and affective factors in human motivation and posits three innate psychological needs that form the basis for optimal self-motivation: competence, relatedness, and autonomy [59]. MI is a client-centered counseling method to explore and resolve uncertainty towards changing behavior and to enhance intrinsic motivation to effect change [68]. SDT and MI have been successfully combined to improve physical activity [69], which has been associated with a common underlying assumption that people have a tendency for personal growth [70,71].

**Figure 2:**
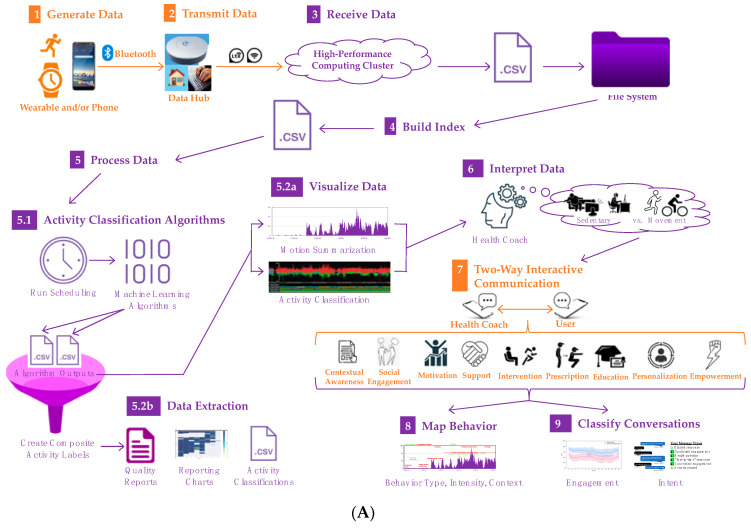

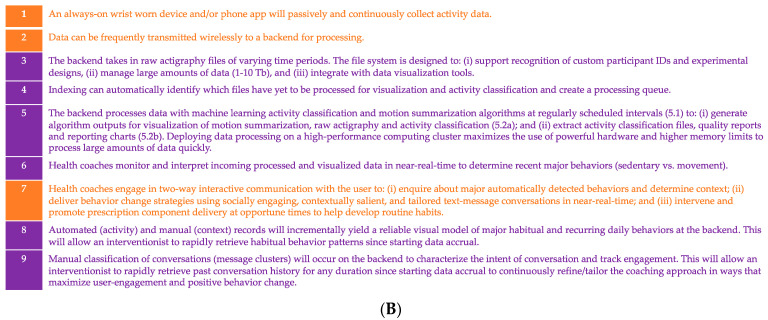
Conceptual model of Companion’s technical components, their integration, and function. (**A**) Flow diagram of the system’s components and their integration; (**B**) function of the system’s components.

*Technology components, their integration, and function:* In general, Companion will comprise a front and backend interface. At the front end, an older adult can experience the Companion through text-based communication using a phone app. An always-on wrist worn device and/or phone app will passively and continuously collect data that can be frequently transmitted wirelessly to a backend for processing to support real-time data gathering on wake and sleep behavior. The phone app will also allow two-way communication and can trigger predetermined messaging (e.g., active CS-EMA [72,73,74]) to actively further data gathering on behavior type, intensity, and its context. The backend interface will be a web-interface that receives processed sensor data and messaging history. Thus, using sensor and conversation data, an interventionist will be able to rapidly retrieve habitual behavior patterns, classify conversations (message clusters) into distinct categories that characterize intent of conversation and engagement rate of the older adult, and retrieve past conversation history for any duration since starting data accrual. All data processing will be conducted on a local server or high-performance computing cluster. Figure 2 displays the conceptual model of Companion’s technical components. Part (A) displays the progression of data gathering, processing, and integration at the front and backend of the Companion system. Front-end and backend interface components are color coded in orange and purple, respectively. Part (B) explains the functions of the system’s individual components.

**Figure 3:**
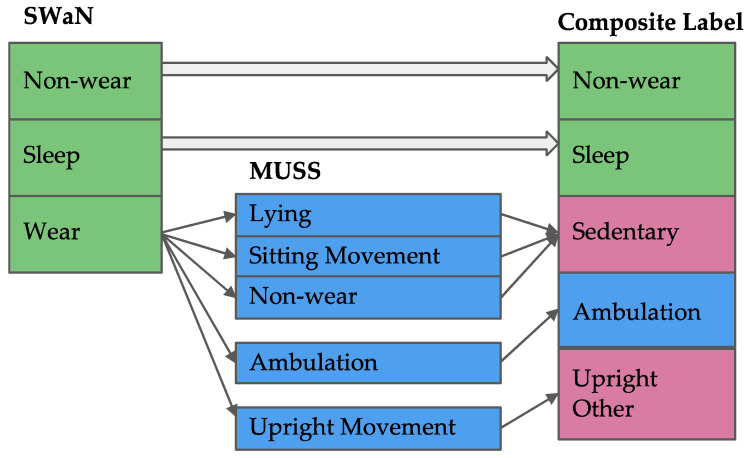
SWaN and MUSS algorithm output merging scheme for generating composite prediction labels. Algorithms outputs are first filtered by SWaN’s non-wear, sleep, and wear classifications. SWaN’s wear event classifications are assigned corresponding to MUSS classifications with lying, sitting movement, and non-wear, which are grouped under a composite “sedentary” label.

**Figure 4:**
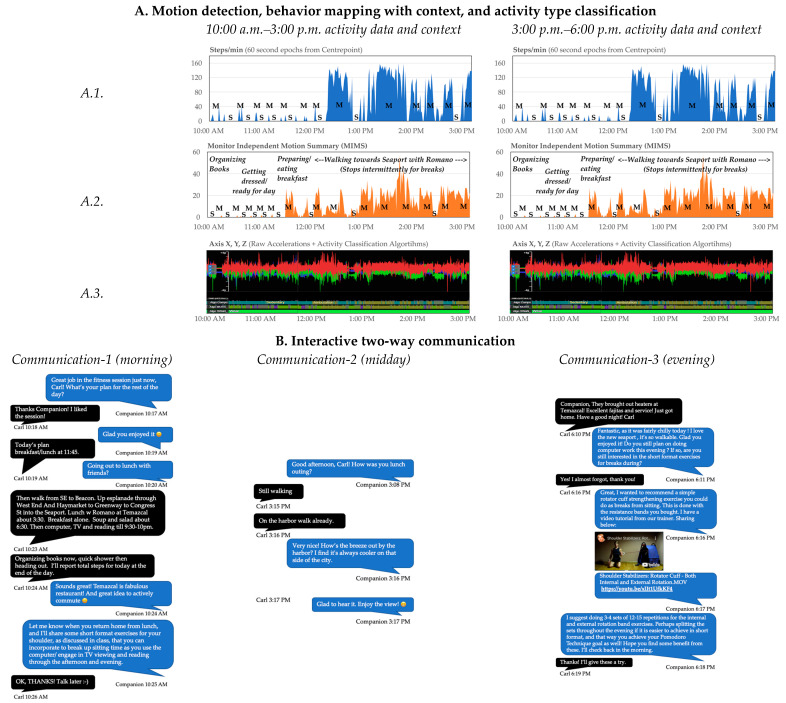
Companion’s personalized and interactive two-way communication, behavior mapping with motion data and context, and activity type classification. (**A**) Health coaches first detect major behaviors (i.e., movement [M] vs. sedentary [S]) by visualizing periods of whole-body locomotion (i.e., steps) on the ActiGraph Centrepoint platform (*A.1*.). This knowledge is used in conjunction with overall wrist motion summary data (MIMS) to further identify periods of non-locomotion movement (*A.2.*), e.g., MIMS helps health coaches identify if periods inferred as sedentary during whole-body locomotion visualization (*A.1*.) are truly sedentary or may involve other types of non-locomotion physical activity. Raw actigraphy data are then processed and visualized on the backend using validated machine learning activity recognition algorithms to classify the signal into selected activity types and non-wear. Such a three-pass data processing and signal visualization strategy yields a reasonable understanding of when an older adult is active and sedentary (*A.3.*). (**B**) Health coaches use interactive two-way text-based communication to enquire about the context of major detected behaviors gathered from activity visualizations (*A.1.*, *A.2.*, and *A.3*.), to further contextualize behavior, and to intervene and promote prescription at opportune times. Three sample conversations at different times of the day (morning, midday, and evening) are depicted here (blue and black boxes indicate Companion’s and the user’s text messages, respectively). Context gathered via text-based communication is overlaid on the backend with periods of sedentariness, locomotion, and other non-locomotion movement to yield a reliable visual model of the context of major behaviors (*A.2.*). Labels (*A.2.*) for major behaviors and *context* are displayed on the lower and upper planes, respectively.

Specific to this pilot, Companion will consist of the following components and workflow: (i) continuously worn wireless wrist sensors (i.e., ActiGraph Insight Watch: triaxial accelerometer; ±8 g; 64 Hz) to capture raw motion data twenty-four hours/day. (ii) These data are transmitted via ActiGraph’s wireless data hubs located at participant homes every hour to ActiGraph’s Centrepoint Platform. (iii) Motion data will be extracted from Centrepoint and processed on our backend located on Northeastern University’s high-performance computing cluster. This backend includes signal visualization software (i.e., Signaligner Pro v2.3.10-beta; Signaligner.org) [42], a signal quality control (QC) algorithm to identify anomalous sensor signals [75] that are unrepresentative of human movement, motion summarization algorithms for total activity volume (i.e., MIMS) [40], and activity classification using machine learning algorithms: (a) SWaN (sleep, wear, and non-wear) [44] and (b) MUSS (i.e., multi-site sensing for activity recognition: lying, sitting movement, non-wear, ambulation, and upright movement) [41,43]. The three algorithmic outputs are up sampled to an event resolution of one second and paired with a composite SWaN plus MUSS prediction (Figure 3). Similar and consecutive one second events with the same neighboring predictions and timestamps are combined into single events, resulting in continuously labeled activity classification events of variable duration. Combining the outputs of SWaN and MUSS into a composite prediction will allow us to retain MUSS’ granularity (ambulation vs. sitting movement vs. upright movement) during wear hours, and SWaN’s more accurate and sophisticated classification of sleep vs. non-wear [41,43,44]. (iv) Processed motion summary and raw sensor data will be visualized by a health coach using the backend interface to temporally display the data with overlaid automatically processed behavior labels. This will enable detection of major behavior types (i.e., movement vs. sedentary) (Figure 4). (v) Health coaches will enquire about major automatically detected behaviors (e.g., movement > 10 min; sedentary > 30 min) to determine context (e.g., walk during lunch; in a regular weekly meeting) using interactive two-way communication. (vi) They can also choose to determine behavior context of any other specific automatically detected behavior. (vii) These automated and manual records will incrementally yield a reliable visual model of major habitual and recurring daily behaviors at the backend. Such information will be richer than what was previously available to an interventionist. (viii) Based on the model, and incremental information being received in near-real-time, coaches will engage the participant via personalized text message conversations to intervene and promote prescription component delivery at opportune times (i.e., near-real-time) to help develop routine habits. Back-and-forth exchanges may or may not be instantaneous, as in asynchronous text messaging conversations (i.e., within a few seconds or minutes). Thus, the Companion will typically respond to certain activities or participant inquiries within minutes, which will maintain the contextual saliency of any information exchange and allow for a just-in-time adaptive intervention. Figure 4 depicts Companion’s personalized and interactive two-way communication, behavior mapping of typical behaviors with motion data and context, and algorithmic activity type classification; data depicted were generated during the pre-study testing phase.

*Messaging strategy*: Communication during weeks 1 to 4 will focus on gathering information on the participant’s motivations, preferences, habits, contexts, and usual behavior patterns to build a typical individual behavior model (Figure 4). Based on our feasibility testing, we expect a decreasing level of change in behavior models that are built using information accrued incrementally over these first 4 weeks.

During weeks 5–16, Companion will monitor ongoing and recent behavior on an hourly basis to process and interpret incoming passive sensor data, as necessary (i.e., there may be delays in sensor data availability when the participant is not home), and periodically communicate with the participant, as necessary. We anticipate engaging with the participant four to seven times/day at intervals of one to four hours to deliver contextually salient intervention strategies. Such frequent contact will enable a highly adaptive and tailored intervention that targets the whole day. The trainers leading the supervised exercise sessions will serve as the health coaches facilitating Companion’s communications, which will (i) allow Companion to be perceived as an extension of the trainer and thus as a valued relationship to foster a sense of accountability in the older adult; (ii) allow continued conversational engagement with a consistent tone/context; and (iii) greatly enhance Companion’s ability to leverage participant motivation during supervised exercise to extend outside periods of supervision. There will be no communications via Companion during the twice/week supervised exercise sessions as trainers will be accessible then.

Initiating communication can work in two ways: Companion will proactively check in with the user by sending a text message, and alternatively, the user may initiate a conversation by sending text messages at any time of day. Text messaging will not involve rigid queries but will rather be open-ended. Additionally, there will be a daily Companion-initiated communication in the early evening (~4 to 6 pm) to discuss the achievement of that day’s goal and prescribe strategies to achieve pending goals. The nature of the first communication the next morning will be based on participant performance during all waking hours of the preceding day, e.g., encouraging, problem solving, etc. Regular interaction will enable a corresponding incremental personalization of communication based on knowledge of each participant’s communication style and personality. Similarly, such frequent interaction will also enable an incremental knowledge of the participant’s changing physical ability and activity preferences, which will facilitate a corresponding increase in personalizing activity prescription.

#### 2.5.4. Exercise Prescription Database

To further facilitate the older adult’s perception of Companion as an extension of the trainer outside periods of supervision, we have compiled a video library of brief (~2–10 min) exercise tutorials available online (i.e., “Companion Study: Exercise Prescription Database”; https://www.youtube.com/channel/UCZsg8fPtquiVrsv8INIvC0g (accessed on 11 November 2022)) [67]. These are categorized by (i) static and dynamic stretches; (ii) aerobic exercises; (iii) flexibility exercises; (iv) upper, lower, and full body resistance exercises; (v) balance exercises; (vi) core and back rehabilitative/resistance exercises; and (vii) shoulder/upper body rehabilitative exercises. Videos from this database will be embedded within the message thread and will enable health coaches to deliver timely but demonstrable “micro-doses” of physical activity and exercise prescriptions that (i) are an extension of age-appropriate exercises prescribed during supervision; (ii) are tailored to the participant’s needs; and (iii) promote “moving more and sitting less” outside supervised sessions. Since this library is not intended to be a comprehensive compilation of exercise and/or fitness tutorials, health coaches may periodically reference external resources, as necessary, based on the participant’s needs, for alternate exercise modes (e.g., yoga, Tai Chi, Pilates, aerobic Tabata, high intensity interval training, etc.), and/or exercise prescriptions for other musculoskeletal rehabilitative needs (e.g., sciatica, hip and/or back pain, plantar fasciitis, etc.). Externally sourced materials (e.g., videos, written exercise tutorials, etc.) will also be embedded within the message thread.

### 2.6. Sample Size and Power Analysis

Sample size calculation was based on conservative power estimates derived using change in daily sedentary behavior among participants (N = 12) in our prior intervention [76], which recruited similarly sedentary individuals (i.e., middle-aged overweight and/or obese seated office workers) and delivered intervention components that encouraged “moving more and sitting less.” This study was a behavior change study using treadmill desks, which although not similar to Companion in terms of intervention delivery, had similar characteristics of being “always available” and a “move more, sit less” behavior change goals. Study arms with at least 17/group were determined to provide 85% power (5% two-tailed significance) to detect an effect size of 1.06 between groups. A standard deviation of 2.8 hours/day for change in total sedentary behavior observed in our prior intervention corresponds to a mean detectable difference of −2.96 sedentary hours/day between groups. Our sample size of 23/group accounts for an attrition rate of 26%.

### 2.7. Planned Data Analyses

All analyses will be conducted using SAS 9.4. (SAS Institute Inc., Cary, NC, USA).

#### 2.7.1. Analyses of Primary and Exploratory Outcomes

Primary outcome variables are (i) daily volume of physical behaviors (i.e., sleep hours/day, wake-time lying hours/day, sitting for seated transport hours/day, other sitting hours/day, standing hours/day, and stepping hours/day and step counts/day) and (ii) physical behavior patterns (e.g., sedentary-to-upright transitions/day, hours/day spent in prolonged [i.e., >30 min] [77] vs. non-prolonged sedentary behavior [78,79]).

Exploratory health outcome variables are (i) blood pressure; (ii) waist and hip circumference; (iii) body mass; (iv) percent body fat; (v) fasting glucose, total cholesterol, and low- and high-density lipoproteins and triglycerides; (vi) age-adjusted percentile scores for executive function, attention skills, episodic memory, working memory, and processing speed; (vii) SPBB score and mean gait speed and chair stand time; (viii) six-minute walk test distance; (ix) maximal number of push-ups and squats completed; (x) and questionnaire outcomes (e.g., from dietary and psychosocial assessment questionnaires).

We will use random intercept mixed linear models, accounting for repeated measures, to assess between- and within-group effects for the primary and exploratory outcomes from baseline to follow-up (after 16 and 24 weeks). Post-hoc pairwise comparisons will be adjusted for multiple comparisons using a Bonferroni correction, and Cohen’s d effect sizes [80] will be calculated for each post-hoc comparison. Data checks will ensure that the underlying assumptions of statistical modeling are not violated by the data, and we will test for potential confounding (i.e., baseline between-group differences in outcome variable and/or demographic compositions) to adjust models accordingly. Losses to follow-up will be handled as intent-to-treat and missing data attributable to unsystematic factors (i.e., missing at random) will be imputed using joint multiple imputation [81,82]. Sensitivity analyses (primary outcomes) will consist of complete-case analyses through 16- and 24-week follow-ups to determine if the treatment responses are altered when examining an ideal but less conservative scenario of complete intervention compliance and to evaluate the sensitivity of the results to the handling of missing data (if applicable) and covariate adjustment (if applicable) [83].

#### 2.7.2. Exploratory Analyses of Intensive Longitudinal Physical Behavior Measurement

We will use multi-group latent growth curve modeling [84,85,86] to estimate the rate and pattern of behavior change over time during the intervention and post-intervention follow-up. These analyses will examine group differences for overall activity volume (mean daily MIMS) and time spent in different activity types (i.e., sleep, sedentary, ambulation, and other upright behaviors). Between-group differences in mean and variance parameters of these variables will be examined after every week. These analyses will characterize (i) how typical individual physical behaviors in the treatment and control conditions change over time, (ii) how much people differ from one another in their physical behavior change within the treatment and control conditions, respectively, and (iii) how typical individual change responses within groups differ between the treatment and control conditions. We hypothesize that the treatment group will show greater individual and group-level positive growth trajectories up to week 8 for total volumes of weekly activity and ambulatory and upright behaviors and negative growth trajectories for total volume of sedentary behaviors. This will be followed by a plateau between weeks 8 and 16 due to increasing stabilization in habitual behavior patterns.

#### 2.7.3. Exploratory Analyses of Intensive Longitudinal Two-Way Communication Data

The goals of these analyses are (i) to create a pool of analytic strategies that guide incremental automation of Companion messaging to enable the scalability of real-world deployment and (ii) to empirically determine strategies that maximize engagement and behavioral responses. To achieve this, we will perform retrospective analyses of Companion–user interactions to determine actionable communication types that maximize Companion–user engagement, and which yield positive behavior change.

*Engagement with Companion:* This will be measured using metrics of receptivity to near-real-time mobile–health interventions: (i) just-in-time response rate (i.e., fraction of Companion-initiated messages to which a user responds within 10 min), (ii) overall response rate (i.e., fraction of all Companion-initiated messages to which the user responds), (iii) median response delay (i.e., time between a Companion-initiated message and the user’s first reply to it), (iv) conversive engagement rate (i.e., fraction of Companion-initiated messages that results in a “conversation”, i.e., a user responds multiple times in a 10 min window after the first Companion-initiated message), and (v) unsolicited engagement rate (i.e., user initiates a message) [87,88]. In addition, we will stratify Companion-initiated messages based on its objective (e.g., educational, goal setting, positive reinforcement, etc.) and tone (e.g., inquisitive, supportive/empowering, social, etc.) (Supplemental Table S1), which will enable the characterization of messages that maximize Companion–user engagement [87,88,89,90,91]. We will use time-varying effect models [92,93,94] to examine changes in gross and stratified Companion–user engagement metrics in the treatment group over the course of the intervention. Time-varying effect models are suitable for analyses of such intensive longitudinal data as these methods can handle missing data and unequal spacing between observations. Engagement will be modeled semi-parametrically as a function of time to examine possible non-linear changes in engagement [95]. We hypothesize that engagement will increase incrementally up to week 8 and plateau/decrease between weeks 8 and 16. The latter will be attributable to increasing stabilization in habitual behavior patterns and increased participant independence.

*Relationship between Companion communication and longitudinal behavior change*: We will examine the time-varying week-to-week relationship between engagement with Companion (total and stratified metrics) and physical behavior outcomes (i.e., mean daily MIMS and volumes of sleep, sedentary, ambulation, and other upright behaviors) using time-varying effect models with an added statistical interaction term in the model that captures the strength of association between engagement and physical behaviors. Since this association can vary across time, changes in the coefficient of the interaction term would (i) highlight periods where program engagement is particularly necessary for ensuring successful behavioral outcomes and (ii) demonstrate the extent to which the relationship between engagement with Companion and positive physical behaviors is sustained.

## 3. Discussion

In this NIH Stage 1 RCT, we design and test the impact of supplementing a traditional supervised exercise program with Companion, a daily near-real-time and interactive two-way communication adaptive behavior change system, on physical behaviors of older adults. The study will recruit 46 overweight and/or obese and sedentary older adults from the Boston community and measure the impact of Companion-based tailored coaching on “moving more and sitting less” outside supervised exercise training as compared to supervised exercise alone.

Our approach has two major advantages over existing mobile health (mHealth) physical activity interventions. (i) Companion will capitalize on advancements in mHealth technologies (e.g., passive wearable physiological sensing, smartphones, cloud-computing, and machine learning algorithms) to extend the known benefits of trainer contact to periods beyond supervised sessions, i.e., the free-living environment of an older adult. (ii) Companion’s human-in-the-loop approach will enable the delivery of socially desirable human traits such as trustworthiness, empathy, attentiveness, compassion, respect, and knowledgeability, all of which may elicit responsiveness against a hesitancy to change. We further discuss these two strengths.

Firstly, our system will have the advantage of allowing users to experience Companion as their “intelligent” human trainer providing extended and on-demand behavior change support outside of face-to-face contact by delivering intervention components and behavior change strategies using socially engaging, contextually salient, and tailored text message conversations in near-real-time. This may motivate older adults to habitually “move more and sit less” and sustain positive behaviors to further improve health. It has been suggested that the most effective technologies for physical activity behavior change may be those that closely match the “gold standard” of frequent and meaningful face-to-face counseling with a trainer [96,97,98] who can communicate the “*right* thing at the *right* time in the *right* way” to the individual [99]. Therefore, a context-sensitive approach that infers human intent and provides socially and motivationally relevant cues may be needed to successfully prompt individuals to act when on their own. While technology-based strategies to improve free-living physical activity are available [100,101,102], most strategies that provide a cue after detecting behavior are one-way feedback systems (e.g., haptic or vibrotactile cue). Such systems place the burden of initiative on the user. User inaction results in wasted opportunities to influence behavior. Thus, one-way feedback systems rapidly become ineffective and cause poor adherence to change [103]. By engaging users in interactive and intelligent two-way communication to deliver behavior change strategies, Companion-like systems may improve the effectiveness of current mHealth behavior interventions. Importantly, two-way communication and information on individual preferences/behaviors better aid users in problem solving, which increases the likelihood of adherence to physical activity in older adults [104,105]. Unlike problem solving with a personal trainer that is typically limited to live supervised sessions, Companion will allow engagement several times daily that can be initiated by the trainer or trainee. This may produce sustainable physical activity habits among sedentary adults outside periods of supervision.

Relatedly, the ability to deliver on-demand behavior change strategies enabled by a mobile technology-driven approach means that Companion-like systems may be more far-reaching to sedentary older adults in need of such interventions (e.g., chronically ill or at-risk populations) than face-to-face strategies. There is already a rapid growth of mobile technology and smartphone use adoption among older adults [106], and the share of the population using mobile technologies is only going to increase as current generations age, e.g., smartphone adoption among those 65 and older in the US population has increased from 13% to 61% in the last decade [106]. This gains further relevance as older adults now represent the fastest growing sector of the population in most economically developed or developing nations [107]. Thus, the necessity for Companion-like systems to reach sedentary older adults and effectively produce sustainable physical activity habits that can impart positive aging effects will continue to grow. Subsequently, the aging Gen X and Millennial generations may also be more comfortable with using mHealth technologies as compared to older adults today (i.e., Baby Boomer generation) because mobile technologies have been more ubiquitous in their lives. Thus, Companion-like support systems may reach a larger proportion of sedentary adults in the future. Importantly, interactive problem solving with a human may be too complex to be executed by even the best computational reasoning systems [108], due to the need for a level of intelligence that may be currently unattainable even with most existing sophisticated AI systems. Our rich intensive longitudinal dataset, comprising four months of daily two-way communications and automated sensor-based behavior monitoring, may enhance the understanding of time-varying effects and mechanisms of behavior change in response to personalized near-real-time mHealth interventions. This may further the understanding of the elements of mHealth interventions that may be automatable and yield an understanding of the scalability of such systems.

Secondly, the frequent contact and personalization that Companion-like systems can offer have the advantage of exploiting the power of social connectedness in daily life. This may (i) help better build rapport, (ii) allow the adult to perceive the virtual Companion as an objectifiable and valued relationship with desirable human traits, and (iii) elicit actionable behavior change leading to innate physical activity habits. Establishing social connectedness in physical activity interventions is particularly important among older adults because aging results in shrinking social networks, more difficulty maintaining social ties, and thus perceptions of low companionship [109]. Importantly, perceived low companionship is associated with increased risk for physical activity declines [110], leading to early morbidity and mortality [111], increased risk for mental health deterioration (e.g., including cognitive decline) [112], poor sleep [112], and cardiovascular health [113]. Therefore, increasing perceived companionship may lead to better and more sustainable health behaviors. However, maintaining social ties to avoid perceptions of low companionship is more difficult in late adulthood [109]. In addition, the last two decades have seen a great decrease in face-to-face “social groups and structures” coupled with an “exponential growth in the use of social media and virtual communication” as a means of social connectedness [114,115,116]. As such, Companion-like systems that leverage mHealth technologies to foster social connectedness and create positive social and behavioral influences may present a new way forward to adapt health interventions to the rapidly shifting landscape of social interaction.

Given these highlighted strengths of our approach, Companion-like systems may positively impact the disease burden associated with the elderly and aging US population. The proportion of US older adults with two or more chronic diseases grew from 66% in 2003 to 83% in 2013 [117]. It is projected that by 2030 there will be an additional 27 million Americans with hypertension, and 12 million with coronary heart disease and stroke [118]. Additionally, the prevalence of neurodegenerative diseases such as Alzheimer’s and Alzheimer’s-related disease are expected to increase by 66% [119,120]. It is well known that an active lifestyle and reduced sedentary behavior attenuates the burden of chronic diseases in older adults including that of neurodegenerative diseases [121]. Yet, despite known benefits, objectively measured data suggest that only 6% of older adults engage in the recommended levels of daily physical activity [32]. While participation in supervised exercise programs is popular in older adults [3,4,5,6,7,8,122], there is evidence that on days of supervised exercise, older adults reduce daily leisure-time walking, and that after commencing an exercise program, movement on non-training days drops below levels prior to program commencement [11,123]. The positive effect of trainer–trainee interaction during supervised exercise needs to be leveraged and transferred beyond periods of supervision. However, it is challenging to maintain the continued and meaningful contact outside sessions that is needed to produce even small transfer effects [124,125,126]. mHealth technology can facilitate this.

This study aims to address limitations of the supervised training model by putting forth and establishing proof-of-concept for an innovative intelligent two-way interaction mobile-intervention approach to motivate older adults to “move more and sit less.” Establishing such proof-of-concept will yield insights that can be used to generate new hypotheses to be tested via larger independently funded NIH Stage 2 RCTs (i.e., traditional efficacy testing), with our long-term aim in mind to deploy a hybrid AI system that elevates scalability and reach to positively impact the ever-growing public health epidemic of sedentariness contributing to poor health outcomes, reduced quality of life, and early death.

## Figures and Tables

**Table 1:** General training progression model.

**Weeks 1–3**	*Introduction, core component education, technique boot camp, and strength foundation*
**Weeks 4–10**	*Incorporation of learned exercises into routines and establishing practical progression benchmarks*
**Weeks 11–16**	*Increasing intensity of routines with a focus on high-interval training and appropriate overloading techniques for power and muscle mass*

## Data Availability

No new data were created or analyzed in this study. Data sharing is not applicable to this article.

## Supplementary Materials

The following supporting information can be downloaded at: https://www.mdpi.com/article/10.3390/s23042221/s1, Study Protocol and Supplemental Table S1. Categorization of Companion and User Messages by Objective and Tone [1,2,3,4,5,6,7,8,9,10,11,12,13,14,15,16,17,18,19,20,21,22,27,28,31,76,127,128,129].

## Abbreviations

AI	Artificial Intelligence
MIMS	Monitor-Independent Movement Summary Unit
SDT	Self-Determination Theory
ACSM	American College of Sports Medicine
MI	Motivational Interviewing
QC	Quality Control
SWaN	Sleep, Wear, and Non-wear
MUSS	Multi-Site Sensing for Activity Recognition
mHealth	Mobile Health
