# Supplementary material for: Companion: A Pilot Randomized Clinical Trial to Test an Integrated Two-Way Communication and Near-Real-Time Sensing System for Detecting and Modifying Daily Inactivity among Adults >60 Years—Design and Protocol
Source: Sensors (Basel). 2023 Feb 16;23(4):2221. doi: 10.3390/s23042221 (PMC9965440; doi:10.3390/s23042221)
Supplement: Supplementary file 1 [file sensors-23-02221-s001.zip › Companion Protocol Document.pdf]

**An Integrated Two-Way Communication and Near-Real-Time Sensing System to Detect and Modify Daily Inactivity among Adults Over Age 60**

**Protocol Number:** CompRoy1

**Principal Investigator:** Dinesh John

**Sponsor:** Northeastern University

**Grant Title:** An Integrated Two-Way Communication and Near-Real-Time Sensing System to Detect and Modify Daily Inactivity among Adults Over Age 60

**Grant Number:** 5P30AG048785-07

**Funded by:** National Institute on Aging

**Version Number:** ver. 2020-9-23

**23 September 2020**

**CONFIDENTIALITY STATEMENT**

This document is confidential communication. Acceptance of this document constitutes agreement by the recipient that no unpublished information contained herein will be published or disclosed without prior approval of the Principal Investigator or other participating study leadership and as consistent with the NIH terms of award.

**Table of Contents**

STATEMENT OF COMPLIANCE ........ 1
INVESTIGATOR'S SIGNATURE ........ 2
1 PROTOCOL SUMMARY ........ 3
1.1 Synopsis ........ 3
1.2 Schema ........ 4
1.3 Schedule of Activities ........ 6
2 INTRODUCTION ........ 7
2.1 Study Rationale ........ 7
2.2 Background ........ 7
2.3 Risk/Benefit Assessment ........ 8
2.3.1 Known Potential Risks ........ 8
2.3.2 Known Potential Benefits ........ 8
2.3.3 Assessment of Potential Risks and Benefits ........ 8
3 OBJECTIVES AND ENDPOINTS ........ 8
4 STUDY DESIGN ........ 9
4.1 Overall Design ........ 9
4.2 Scientific Rationale for Study Design ........ 10
4.3 Justification for Intervention ........ 10
4.4 End-of-Study Definition ........ 10
5 STUDY POPULATION ........ 10
5.1 Inclusion Criteria ........ 11
5.2 Exclusion Criteria ........ 11
5.3 Lifestyle Considerations ........ 11
5.4 Screen Failures ........ 11
5.5 Strategies for Recruitment and Retention ........ 11
6 STUDY INTERVENTION(S) OR EXPERIMENTAL MANIPULATION(S) ........ 12
6.1 Study Intervention(s) or Experimental Manipulation(s) Administration ........ 12
6.1.1 Study Intervention or Experimental Manipulation Description ........ 12
6.1.2 Administration and/or Dosing ........ 15
6.2 Fidelity ........ 15
6.2.1 Interventionist Training and Tracking ........ 16
6.3 Measures to Minimize Bias: Randomization and Blinding ........ 16
6.4 Study Intervention/Experimental Manipulation Adherence ........ 16
6.5 Concomitant Therapy ........ 16
6.5.1 Rescue Therapy ........ 16
7 STUDY INTERVENTION/EXPERIMENTAL MANIPULATION DISCONTINUATION AND PARTICIPANT DISCONTINUATION/WITHDRAWAL ........ 16
7.1 Discontinuation of Study Intervention/Experimental Manipulation ........ 17
7.2 Participant Discontinuation/Withdrawal from the Study ........ 17
7.3 Lost to Follow-Up ........ 17
8 STUDY ASSESSMENTS AND PROCEDURES ........ 17
8.1 Endpoint and Other Non-Safety Assessments ........ 17

8.2 Safety Assessments ... 23
8.3 Adverse Events and Serious Adverse Events ... 23
8.3.1 Definition of Adverse Events ... 23
8.3.2 Definition of Serious Adverse Events ... 23
8.3.3 Classification of an Adverse Event ... 23
8.3.4 Time Period and Frequency for Event Assessment and Follow-Up ... 24
8.3.5 Adverse Event Reporting ... 24
8.3.6 Serious Adverse Event Reporting ... 25
8.3.7 Reporting Events to Participants ... 25
8.3.8 Events of Special Interest ... 25
8.3.9 Reporting of Pregnancy ... 25
8.4 Unanticipated Problems ... 25
8.4.1 Definition of Unanticipated Problems ... 25
8.4.2 Unanticipated Problems Reporting ... 25
8.4.3 Reporting Unanticipated Problems to Participants ... 26
9 STATISTICAL CONSIDERATIONS ... 26
9.1 Statistical Hypotheses ... 26
9.2 Sample Size Determination ... 26
9.3 Populations for Analyses ... 26
9.4 Statistical Analyses ... 26
9.4.1 General Approach ... 26
9.4.2 Analysis of the Primary Endpoint(s) ... 27
9.4.3 Analysis of the Secondary Endpoint(s) ... 27
9.4.4 Safety Analyses ... 27
9.4.5 Baseline Descriptive Statistics ... 27
9.4.6 Planned Interim Analyses ... 27
9.4.7 Sub-Group Analyses ... 27
9.4.8 Tabulation of Individual Participant Data ... 27
9.4.9 Exploratory Analyses ... 28
10 SUPPORTING DOCUMENTATION AND OPERATIONAL CONSIDERATIONS ... 28
10.1 Regulatory, Ethical, and Study Oversight Considerations ... 28
10.1.1 Informed Consent Process ... 28
10.1.2 Study Discontinuation and Closure ... 28
10.1.3 Confidentiality and Privacy ... 29
10.1.4 Future Use of Stored Specimens and Data ... 30
10.1.5 Key Roles and Study Governance ... 30
10.1.6 Safety Oversight ... 30
10.1.7 Clinical Monitoring ... 31
10.1.8 Quality Assurance and Quality Control ... 31
10.1.9 Data Handling and Record Keeping ... 31
10.1.10 Protocol Deviations ... 32
10.1.11 Publication and Data Sharing Policy ... 33

10.1.12 Conflict of Interest Policy .......... 33
10.2 Additional Considerations .......... 33
10.3 Abbreviations and Special Terms .......... 34
10.4 Protocol Amendment History .......... 35
11 REFERENCES .......... 37

## STATEMENT OF COMPLIANCE

The trial will be conducted in accordance with International Council on Harmonization Good Clinical Practice (ICH GCP), applicable United States (US) Code of Federal Regulations (CFR), and the NIA Terms and Conditions of Award. The Principal Investigator will assure that no deviation from, or changes to the protocol will take place without prior agreement from the funding agency and documented approval from the Institutional Review Board (IRB), except where necessary to eliminate an immediate hazard(s) to the trial participants. All personnel involved in the conduct of this study have completed Human Subjects Protection and ICH GCP Training.

The protocol, informed consent form(s), recruitment materials, and all participant materials will be submitted to the IRB for review and approval. Approval of both the protocol and the consent form(s) must be obtained before any participant is consented. Any amendment to the protocol will require review and approval by the IRB before the changes are implemented to the study. All changes to the consent form(s) will be IRB approved; a determination will be made regarding whether a new consent needs to be obtained from participants who provided consent, using a previously approved consent form.

## INVESTIGATOR'S SIGNATURE

The signature below constitutes the approval of this protocol and provides the necessary assurances that this study will be conducted according to all stipulations of the protocol, including all statements regarding confidentiality, and according to local legal and regulatory requirements and applicable US federal regulations and ICH guidelines, as described in the Statement of Compliance above.

Principal Investigator or Clinical Site Investigator:

Signed: 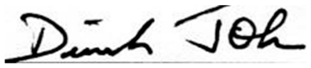 Date: 4-27-2021

Name*: Dinesh John

Title*: Associate Professor

Investigator Contact Information
Affiliation*: Northeastern University
Address: 316 Robinson Hall, 360 Huntington Ave., Boston, Massachusetts
Telephone: 617373-5695
Email: d.john@northeastern.edu

# 1 PROTOCOL SUMMARY

## 1.1 SYNOPSIS

| | |
|---|---|
| **Title:** | An Integrated Two-Way Communication and Near-Real-Time Sensing System to Detect and Modify Daily Inactivity among Adults Over Age 60 |
| **Grant Number:** | 5P30AG048785-07 |
| **Study Description:** | The objective is to test if Companion, an Integrated Two-Way Communication and Near-Real-Time Sensing System, improves and sustains overall daily free-living activity and sedentary behavior in adults over 60 y. The central hypothesis is that Companion will improve overall daily free-living activity and sedentary behavior in adults>60 y and thereby improve their health. |
| **Objectives*:** | Primary Objective: Determine if Companion improves free-living activity and sedentary behavior and sustains this change in adults >60y. In this RCT (n=46), both treatment and control groups will engage in a 16-week supervised exercise program and then be followed-up after 24 weeks.[1,2] Companion will be deployed in the treatment group (n=23) only. Primary outcomes are daily total and patterns of physical activity and sedentary time.<br><br>Exploratory Objective: Explore if supplementing supervised exercise with Companion further improves and sustains cardiometabolic health, body composition, cognition, and psychosocial outcomes. |
| **Endpoints*:** | Primary Endpoint: Physical activity and sedentary time: Daily total and patterns<br><br>Exploratory Endpoints: Anthropometrics; Plasma glucose; HbA1C; Insulin; Lipids; Lean and fat mass; Physical function; Muscular and aerobic fitness; Cognition; perceptions of competence related to health behavior; Autonomy support (from companion); Autonomous motivation (for physical activity); Relatedness (to the Companion); General satisfaction. |

| | |
|---|---|
| **Study Population:** | N= 46, gender- at least 50% female, age>60 y, demographic group- older adults; general health status- healthy; geographic location- Boston and surrounding neighborhoods. |
| **Phase* or Stage:** | 1A |
| **Description of Sites/Facilities Enrolling Participants:** | The Human Performance and Exercise Science (HPES) Laboratory is located in the Behrakis Health Science Center at Northeastern University, which will be the sole site for the study. Participants who do not prefer to have their measurements conducted at their residence will come to the lab to have measurements completed. The study is not intended to include sites outside of the United States. |
| **Description of Study Intervention/Experimental Manipulation:** | The study intervention consists of wearable sensors integrated with the smartphone to develop a behavior-aware, virtual “Companion” that uses a “human-in-the-loop” approach to enable meaningful two-way communication. Companion builds rich models of typical behavior using sensors and interactive two-way communications to deliver intervention components and behavior change strategies using socially engaging, contextually salient, and tailored text-message conversations in near-real-time. The duration of the intervention is 4 months. |
| **Study Duration*:** | Estimated time (in months) from when the study opens to enrollment until completion of data collection is 6.5 months per participant. We anticipate a rolling enrollment with approximately 3 waves, resulting in an estimated timeframe of 19.5 months from when the study opens to enrollment and completion of data collection for all subjects. |
| **Participant Duration:** | Time for each individual participant to complete all study-related tasks is 6.5 months. |

## 1.2 SCHEMA

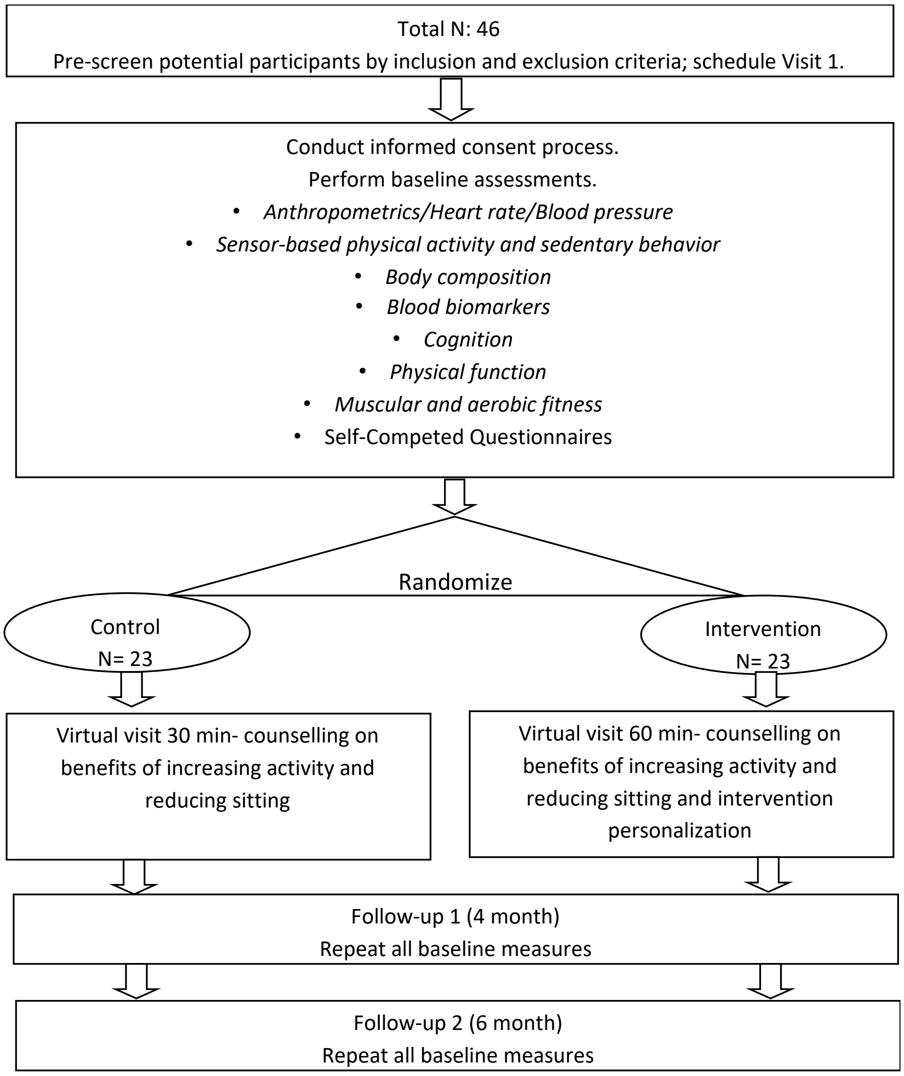
Total N: 46
Pre-screen potential participants by inclusion and exclusion criteria; schedule Visit 1.
Conduct informed consent process.
Perform baseline assessments.
• Anthropometrics/Heart rate/Blood pressure
• Sensor-based physical activity and sedentary behavior
• Body composition
• Blood biomarkers
• Cognition
• Physical function
• Muscular and aerobic fitness
• Self-Competed Questionnaires
Randomize
Control
N= 23
Intervention
N= 23
Virtual visit 30 min- counselling on benefits of increasing activity and reducing sitting
Virtual visit 60 min- counselling on benefits of increasing activity and reducing sitting and intervention personalization
Follow-up 1 (4 month)
Repeat all baseline measures
Follow-up 2 (6 month)
Repeat all baseline measures

## 1.3 SCHEDULE OF ACTIVITIES

| | Pre-screening (Pre-consent) | Measurement 1 Day 1 | Virtual visit Day 7-14 | Measurement 2 Day 120-130 | Measurement 3 Day 240-250 |
|---|---|---|---|---|---|
| Review Eligibility | X | | | | |
| Informed Consent | | X | | | |
| Demographics | | X | | | |
| Outcome Evaluation | | X | | X | X |
| Randomization | | X | | | |
| Control & Experimental Interventions | | → | → | → | |
| Counselling | | | X | | |
| Adverse Events Reporting | | X | X | X | X |

# 2 INTRODUCTION

## 2.1 STUDY RATIONALE

This work will result in a new approach to extend the efficacy of supervised training beyond brief periods of direct exposure and prolong engagement in positive behaviors after program completion.

## 2.2 BACKGROUND

Supervised exercise with a trained instructor[3,4] is most effective in maximizing health benefits, is popular among adults>60y, and is most common in aging research .[5-10] Supervision results in higher training effects and program adherence[7,8,11,12] due to personal attention and interaction with the trainer that motivate participants.[8,11] Drawbacks of the model include a low frequency of trainer contact (60 min, ~1 to 3 times/week) and ineffectiveness in changing behavior outside weekly sessions.[8] Additionally, adults>60y compensate for increased exercise by increasing sedentary behavior outside training sessions, which attenuates positive gains in health from supervised exercise.[10,13-19] Independent of physical activity, reducing sedentary time improves cardiometabolic and functional health in adults>60y.[20,21] Thus, federal guidelines recommend that older adults “move more and sit less” during the day.[22] Strategies to motivate adults>60y who participate in supervised programs to be more active and sit less outside structured weekly training sessions are necessary.

Technologies including wearables and smartphones have great potential to promote daily positive behaviors in adults>60y.[23] Nonetheless, a significant research gap is that “such technologies rarely integrate meaningful two-way communication,[24]” which allows an interventionist to successfully engage an adult via an intelligent, context-sensitive dialogue to problem-solve and motivate change.[24] Available two-way communication interventions (e.g., text message and internet-based strategies) have limited meaningfulness due to an inability to appropriately time the communication and tailor it based on ongoing or recent behavior.

Awareness of: (i) ongoing or recent behavior (from sensors), (ii) situational contexts of the behavior, (iii) past conversation, and (iv) the adult’s preferences (# ii, iii, and iv gathered via two-way communication) will help generate meaningful discourse that allows the creation of an adaptive intervention at a level that was not previously possible. We have integrated wearable sensors with the smartphone to develop a behavior-aware, virtual “Companion” that uses a “human-in-the-loop” approach to enable meaningful two-way communication. Companion builds rich models of typical behavior using sensors and interactive two-way way communications to deliver intervention components and behavior change strategies using socially engaging, contextually salient, and tailored text-message conversations in near-real-time.

In our feasibility testing (3 m) of Companion, adults>60y (n=6) reduced daily sitting by ~2.1 h and improved health.[25] Independent of disease, an active lifestyle also improves cognition in older adults.[26,27] Thus, an investigation of the impact of Companion on behavioral, cognitive, and physiological outcomes is required.

## 2.3 RISK/BENEFIT ASSESSMENT

### 2.3.1 KNOWN POTENTIAL RISKS

There is a risk of physical injury during exercise, particularly if participants have little to no previous experience. Fatigue and muscle soreness may also occur in participants who do not normally engage in physical activity. Exercise is associated with a very small risk of serious medical complications including heart attack and sudden death, although this risk is particularly small in healthy older adults.

There is a potential risk of breach of confidentiality that is inherent in all research protocols.

Some inconvenience and or anxiety may occur due to time required to complete formal rating scales and questionnaires. The cognitive assessments impose some risk of emotional discomfort. Fatigue associated with performing the cognitive tasks- These items will be scored within 24 hours of completion by the subject.

Participants may experience minor skin irritation or discomfort from wearing sensor devices for an extended period of time.

### 2.3.2 KNOWN POTENTIAL BENEFITS

Increasing physical activity, regular structured exercise, and reducing sedentary behavior, improves cognitive and physiological health.[1-3] Supervised exercise programs (group/individual) involving direct contact with a trainer are most effective in maximizing health benefits in adults >60 y; hence, such programs are popular in adults >60 y and are widely offered by aging-related interest groups.[2,4-8] Supervision results in fewer injuries, higher training effects, and program adherence.[5,6,9,10] Adherence is due to personal attention and interaction between the participant and supervisor during sessions, which motivate the participant.[6,9] mHealth technologies including mobile phones and wearables hold immense potential to engage adults >60 y and promote positive behavior change throughout the day.[21]

### 2.3.3 ASSESSMENT OF POTENTIAL RISKS AND BENEFITS

The risks are minimal and outweighed by the potential benefits to individuals and society
All experimenters involved in the project will be CPR and First Aid certified.

# 3 OBJECTIVES AND ENDPOINTS

| OBJECTIVES | ENDPOINTS | JUSTIFICATION FOR ENDPOINTS |
|---|---|---|
| Primary | | |

| OBJECTIVES | ENDPOINTS | JUSTIFICATION FOR ENDPOINTS |
|---|---|---|
| Determine if Companion improves free-living activity and sedentary behavior and sustains this change in adults >60y. | Daily total and patterns of physical activity and sedentary time. | These physical behaviors are directly impacted by motivation communication aimed at promoting positive behavior change. |
| Tertiary/Exploratory | | |
| (1) Explore if supplementing supervised exercise with Companion further improves and sustains cardiometabolic health, body composition, physical function, muscular and aerobic fitness, and cognition.<br>(2) Examine factors that mediate and moderate change in physical activity and sedentary behavior. | Cardiometabolic health (Plasma glucose, HbA1C, insulin, and lipids; lean and fat mass; Physical function; Cognition; Perceptions of competence related to health behaviors; Autonomy support (from companion); Autonomous motivation (for physical activity); Relatedness (to the Companion); General satisfaction. | These outcomes may be impacted by change in behavior and communication with Companion |

# 4 STUDY DESIGN

## 4.1 OVERALL DESIGN

Hypothesis (Aim 1): The treatment group will show greater improvements in daily activity and sedentary behavior outcomes than controls after 16 and 24 weeks.

Hypothesis (Aim 2): The treatment group will show greater improvements in health and psychosocial

outcomes than controls after 16 and 24 weeks.

Phase: 1A.

Design: Randomized control trial

Participant assignment: 1:1 Simple Randomization conducted by graduate assistant

Number of study group: 2 (control/treatment); intervention period= 4 months; follow-ups after 4 and 6 months.

Number of sites= Single site trial.

Name and description of intervention: Companion; An integrated system of wearable sensors and the smartphone. It is a behavior-aware, virtual "Companion" that uses a "human-in-the-loop" approach to enable meaningful two-way communication. Companion builds rich models of typical behavior using sensors and interactive two-way communications to deliver intervention components and behavior change strategies using socially engaging, contextually salient, and tailored text-message conversations in near-real-time.

Description of control group: This group will not receive the Companion, which will be the only difference with the treatment group; i.e., the control group will receive standard treatment– the supervised training program and an exercise prescription for those days without supervision.

## 4.2 SCIENTIFIC RATIONALE FOR STUDY DESIGN

An RCT design where the control group does not receive the Companion intervention will afford the ability to tease apart the efficacy of the Companion. The design will minimize the risk of confounding, help to increase confidence in the findings, and thus, are more likely to be representative of the true effect of the intervention.

## 4.3 JUSTIFICATION FOR INTERVENTION

The intervention is delivered via text messaging as it is an mHealth intervention that targets behavior improvement when participants are on their own. Participants will receive 4-7 messages from the Companion in intervals of 1-4 hours each day. This limited interval will retain the novelty of the communication and thus interest of the participant in engaging in the study while yielding evaluable data.

## 4.4 END-OF-STUDY DEFINITION

A participant is considered to have completed the study if he or she has completed the baseline assessment and the 4-month follow-up assessment.

# 5 STUDY POPULATION

## 5.1 INCLUSION CRITERIA

1. Provision of signed and dated informed consent form
2. Age >60 y
3. Have a body mass index of greater than or equal to 25 but less than 40 $kg/m^2$
4. No conditions preventing participation in physical activity lasting 10 to 30 min
5. Have a smart phone
6. Physician clearance to participate in moderate-vigorous intensity physical activity

## 5.2 EXCLUSION CRITERIA

1. Engage in structured physical activity for more than 2 days/week lasting 30 min/session-
2. Regularly use any assistive device for walking.
3. Likely to alter medications pertaining to cardiovascular or metabolic health
4. Health conditions (e.g., cardiovascular, metabolic, musculoskeletal) preventing participation in physical activity lasting 10 to 30 min

## 5.3 LIFESTYLE CONSIDERATIONS

NA

## 5.4 SCREEN FAILURES

Screen failures are defined as participants who consent to participate in this study but are not subsequently assigned to the study intervention or entered in the study. Individuals who do not meet the criteria for participation in this trial (screen failure) because of meeting/not meeting one or more exclusion/inclusion criteria, respectively will not be enrolled in the study.

## 5.5 STRATEGIES FOR RECRUITMENT AND RETENTION

Anticipated number of people to be screened =56; Women and Minorities= at least 50 and 20% respectively; Anticipated number of people to be enrolled =46; Women and Minorities= at least 50 and 20% respectively;

Participation in this study will be sought using flyers (see attached flyer) and email (see attached recruitment email template) to identify interested individuals. We will identify potentially eligible Northeastern employees from University department's online staff directories and obtain individual email addresses from employee directories to contact individual's directly in seeking participation in our study using the aforementioned recruitment email and flyer. Recruitment materials (email and flyer) will include the self-inclusion criteria. Should the conditions not apply to individuals who are contacted, he or she may exclude himself or herself without the need to reveal his or her reasons to the researchers. Recruitment emails will be sent a second time a week later if there is no response on the initial invite. People who meet the self-inclusion criteria and express interest in further information will be asked to set up a time to meet briefly at their Northeastern office/home/virtually with the researchers.

This strategy is appropriate because Northeastern University has over 2000 employees engaged in seated work and will yield the necessary sample size for the study. Additionally, Northeastern University is located in close proximity to several other academic institutions such as the Massachusetts College of Arts, Massachusetts College of Pharmacy, Wentworth Institute of Technology, the New England Conservatory of Music, and Harvard School of Public Health and its Medical School. We will direct recruiting efforts at these institutions as well, which are all less that ¼ mile from NU's campus. Additionally, we will reach out to senior centers in Boston and surrounding areas and other resources including social media and listservs that are publicly available.

The targeted distribution of the participants' racial and ethnic makeup is anticipated to be successful given the demographic distribution in the city of Boston based on estimates obtained from United States Census bureau. According to these data, the distribution of minorities in the general population in Massachusetts is 47.4%, Hispanic or Latino of any race is 19.7%, Black or African American alone is 25.3%, American Indian or Alaska native is 0.3%, Asian alone is 9.6%, and those identifying as being of two or more races is 5.1%. White adults are the majority at 52.6%. The proportion of women in the city of Boston is 51.9%.

Participants may receive up to $120 in remuneration, consisting of $40 for completing 3 measurements either at the Human Performance and Exercise Science Lab or in their place of residence. Payment will be provided after completion of the study, either after the month-6 follow up follow-up or at an earlier point should the participant drop out of the study prior to moth 6 follow up, consistent with the measurement completion criteria. The incentive is appropriate and will not be viewed as coercive.

# 6 STUDY INTERVENTION(S) OR EXPERIMENTAL MANIPULATION(S)

## 6.1 STUDY INTERVENTION(S) OR EXPERIMENTAL MANIPULATION(S) ADMINISTRATION

### 6.1.1 STUDY INTERVENTION OR EXPERIMENTAL MANIPULATION DESCRIPTION

The Companion: Companion is based on Self Determination Theory (SDT) and the application of Motivational Interviewing (MI) to creatively use information on behaviors, attitudes, and environmental contexts to create user-Companion interactions that are tailored to participant needs. SDT addresses cognitive and affective factors in human motivation and posits three innate psychological needs that form the basis for optimal self-motivation: competence, relatedness, and autonomy.[30] MI is a client-centered counseling method to explore and resolve uncertainty towards changing behavior and to enhance intrinsic motivation to effect change.[31] SDT and MI are successfully combined to improve physical activity[32] because of a common underlying assumption that people have a tendency for personal growth toward psychological integration.[33,34]

Next, we describe Companion's technological components, their integration, and integrated function: (1) Continuously worn wireless ActiGraph® wrist sensor capture raw motion data. (2) These data are transmitted hourly to a backend. (3) Motion data are processed immediately at the backend using validated machine learning algorithms to characterize data as physical activity (e.g. walking or other) or sedentary. (4) Processed data are visualized by a health coach using a backend interface that temporally displays estimated behavior types. (5) Research assistants will enquire about major automatically detected behaviors (e.g. movement >10 min; sitting >20 min) to determine context (e.g. walk during lunch; in a

weekly meeting) using interactive two-way communication. (6) They can also choose to determine behavior context of any other automatically detected behavior. (7) These automated and manual records will incrementally yield a reliable visual model of major habitual daily behaviors at the backend. Such information will be richer than what was previously available to an interventionist. (8) Based on the model, coaches will use the two-way communication app to intervene and promote prescription component delivery at opportune times (i.e., near-real-time) to help develop routines.

Supervised Exercise Program: For the first 16 weeks, all participants will engage in an age-appropriate supervised exercise program for ~45 to 60 min consisting of aerobic, strength, flexibility and balance training, twice/week (Table 3).[29,35] Participants will either visit Northeastern's Exercise Science Lab for supervised personal training or engage virtually over Zoom during the training session. The lead trainers will be an exercise physiologist or a trained graduate student. After the session, the trainer and participant will outline goals and prescription plan for the days prior the next training session and those to be performed by the participant on their own. The plan will be based on guidelines from the American College of Sports Medicine.[36,37] Supervised training sessions for the two groups will be scheduled on different days and allocation of participants to a trainer will be at random.

**Table 3.** 16 Week Supervised Exercise Training Progression Model

**Weeks 1-3** *(Introduction, core component education, technique boot camp and strength foundation)*

- Moderate-to-vigorous intensity aerobic warmup (i.e., ~60-80% of age predicted max HR and/or RPE to target sustainment and improvement of cardiorespiratory fitness) and dynamic stretching
- Introduction and education on multiple-joint exercises (e.g., squat, deadlift, pushup, row, etc.)
  - Mechanics of exercises, breathing techniques, talk-test, mind-muscle connection, assessing 1RM
- Concentric, eccentric and isometric actions introduced for each exercise
- 40-50% 1RM used for 10-12 repetitions for novice to intermediate trainees
  - 1-3 sets/exercise used for novice individuals
- 50-60% 1RM used for 8-10 repetitions for advanced trainees
  - Multiple-set programs introduced with varying levels of volume and intensity for advanced trainees
- 1-3 min rest in between sets for beginner, intermediate and advanced trainees
- Slow-to-moderate lifting velocity
- Plyometric exercises with zero-to-low resistances introduced for novice trainees (chair and bed-based exercises)
- Balance exercises to improve stability and agility
- Cooldown: static stretching and mobility exercises (increase range of motion and joint flexibility)

**Weeks 4-10** *(Incorporation of learned exercises into routines and establishing practical progression benchmarks)*

- Moderate-vigorous intensity aerobic warmup (i.e., ~60-80% of age predicted max HR and/or RPE to target sustainment and improvement of cardiorespiratory fitness) and dynamic stretching
- Emphasis on multiple-joint exercises
- 60-70% 1RM for 8-12 repetitions for novice to intermediate trainees
- Cycling loads of 60-80% 1RM for advanced trainees
- Progression benchmark: when training at a specific 1RM load, increase the load by 2-10% when the subject can perform 1-2 repetitions over the desired number of repetitions on two consecutive training sessions
- 1-3 min rest in between sets
- Slow-to-moderate lifting velocity
- Balance exercises to improve stability and agility
- Cooldown: static stretching and mobility exercises (increase range of motion and joint flexibility)

**Weeks 11-16** *(Increasing intensity of routines with a focus on high-interval training and appropriate overloading techniques for power and muscle mass)*

- Moderate-vigorous intensity aerobic warmup (i.e., ~60-80% of age predicted max HR and/or RPE to target sustainment and improvement of cardiorespiratory fitness) and dynamic stretching
- Higher intensity interval training (e.g., Tabata) [61]
  - 40 seconds constant work, 20 seconds break, 1 min rest between circuits
  - Shorter rest periods for muscular endurance training
- Progressive overloading techniques (intensifiers)
  - Supersets- one exercise followed by another with no rest in between sets)
    - e.g., Resistance band row, (10 repetitions) followed by resistance band bicep curl (10 repetitions)
  - Drop-sets- one exercise followed by the same exercise with a lower load
    - e.g., 8 standard push-ups followed by 4 push-ups performed on the knees
  - As many repetitions as possible (AMRAP) are performed with proper form being the key consideration
- Increasing strength and hypertrophy
  - Slow-to-moderate lifting velocity, for 1-3 sets per exercise with 60-80% 1RM for 8-12 repetitions with 1-3 min rest periods between sets
- Increasing power
  - High repetition velocity, 30-60% 1RM for 6-10 repetitions
- Increasing muscular endurance

  - Low to moderate loads performed for moderate to high repetitions (10-15 or more)
- Balance exercises to improve stability and agility
- Cooldown: static stretching and mobility exercises (increase range of motion and joint flexibility)

Treatment Group: This group will receive the Companion. Communication during weeks 1 to 4, will focus on gathering information on the participant's motivations, preferences, habits, contexts, and usual behavior patterns to build a typical behavior model for the participant. We expect a decreasing level of change in behavior models that are built using information accrued incrementally over these 4 weeks.[23]

Companion will monitor ongoing and recent behavior, communicate with the participant (if necessary) to deliver contextually salient intervention strategies (i.e., age appropriate exercise and/or physical activity prescriptions). Additionally, there will be a fixed Companion-initiated communication in the early evening (~4 to 6 pm) to discuss the achievement of that day's goals and prescribe strategies to achieve pending goals.

The nature of the first communication the next morning will be based on participant performance during all waking hours of the preceding day- e.g., encouraging, problem-solving. This is possible because the participant will be instructed to continuously wear the wrist device, which stores motion data in its internal memory. These data will be downloaded to Companion and analyzed.

Regular interaction will enable a corresponding incremental personalization of communication based on knowledge of each participant's communication style and personality. Similarly, such frequent interaction will also enable an incremental knowledge of the participant's changing physical ability and activity preferences, which will facilitate a corresponding increase in personalizing activity prescription.

Control Group: This group will not receive the Companion, which will be the only difference with the treatment group; i.e., the control group will receive standard treatment– the supervised training program and an exercise prescription for those days when they are on their own. This will be done on the two days when control group participants meet face-to-face with the trainer for supervised training.

Primary Outcomes: Total duration and number of patterns of: (i) light and moderate-to-vigorous physical activity and (ii) sedentary behavior measured over a period of one week using accelerometry.[38,39] Week-long measures will be made at baseline, and weeks 16 and 24.

Exploratory Outcomes: Cardiometabolic health: Plasma glucose, HbA1C, insulin, and lipids. Body composition: lean and fat mass. Physical function. Muscular and aerobic fitness. Cognition.[42] Perceptions of competence related to health behaviors. Autonomy support (from companion). Autonomous motivation (for physical activity). Relatedness (to the Companion). General satisfaction.

### 6.1.2 ADMINISTRATION AND/OR DOSING

Please see section 6.1.1.

## 6.2 FIDELITY

### 6.2.1 INTERVENTIONIST TRAINING AND TRACKING

Specific to the intervention the table below identifies the frequency of monitoring intervention fidelity:

| Data source | Monitoring frequency | Area of fidelity | |
|---|---|---|---|
| | | Study design | Treatment delivery |
| Personal training checklist | Weekly | x | x |
| Review of two-way messaging | 2 to 4 days | x | x |
| Team meetings to discuss participant progress and protocol adherence | Weekly | x | |

Additionally, prior to commencing the study, research staff will undergo training on how to maintain consistency when communicating via Companion and during outcome measurement. Communication training will be based on guidelines for motivational interviewing. After training on outcome measurement, a skill test will be conducted on a test individual by research staff to determine inter-tester agreement.

## 6.3 MEASURES TO MINIMIZE BIAS: RANDOMIZATION AND BLINDING

1:1 Simple Randomization conducted by graduate assistant.

It has been suggested that blinding in behavioral interventions such as those involving physical activity and exercise is comparatively more challenging than in pharmacological or medical studies; this may be more relevant to pilot trials that are not conducted as part of a larger trial, which aim to assess the conceivable benefits of an intervention and the potential of conducting a future definitive RCT.[7-9] While the health coaches and participants will not be blinded to treatment allocation, we minimize bias via the use of objectively measured outcome variables in both aims. Furthermore, the statistical analyst will be blinded to treatment allocation during analyses.

## 6.4 STUDY INTERVENTION/EXPERIMENTAL MANIPULATION ADHERENCE

Adherence to the study intervention will be monitored using multiple sources of information. These include (1) the frequency of messages sent and received using the Companion, which will be tracked weekly (2) attendance in the personal training sessions, which will be tracked weekly, and (3) outcome assessment attendance, which will be tracked at the end of the study.

## 6.5 CONCOMITANT THERAPY

### 6.5.1 RESCUE THERAPY

NA

# 7 STUDY INTERVENTION/EXPERIMENTAL MANIPULATION DISCONTINUATION AND PARTICIPANT DISCONTINUATION/WITHDRAWAL

## 7.1 DISCONTINUATION OF STUDY INTERVENTION/EXPERIMENTAL MANIPULATION

The criteria for discontinuing the intervention include the participant's request, as well as any life-threatening or potentially disabling event, including acute illness, an injurious non-accidental fall, or hospitalization. These adverse events will be recorded in accordance with the Northeastern IRB requirements.

## 7.2 PARTICIPANT DISCONTINUATION/WITHDRAWAL FROM THE STUDY

The criteria for discontinuing the intervention include the participant's request, as well as any life-threatening or potentially disabling event, including acute illness, an injurious non-accidental fall, or hospitalization. These adverse events will be recorded in accordance with the Northeastern IRB requirements.
Additionally, an investigator may discontinue a participant if:

- Lost-to-follow up; unable to contact subject.
- The participant meets an exclusion criterion (either newly developed or not previously recognized) that precludes further study participation

The reason for participant discontinuation or withdrawal from the study will be recorded.
Participants who sign the informed consent form and are randomized but do not receive the study intervention may be replaced. Participants who sign the informed consent form, are randomized and receive the study intervention, and subsequently withdraw or are discontinued from the study, will not be replaced.

## 7.3 LOST TO FOLLOW-UP

A participant will be considered lost to follow-up if he or she fails to return for both follow-up measurements and if study staff are unable to contact the participant after at least 3 attempts.

If a participant fails to be available for a follow-up:

- The researchers will attempt to contact the participant, reschedule the missed measurement with a week, counsel the participant on the importance of maintaining the measurement schedule and ascertain if the participant wishes to and/or should continue in the study.
- Before a participant is deemed lost to follow-up, the investigator or designee will make every effort to regain contact with the participant (where possible, 3 telephone calls and, if necessary, a certified letter to the participant’s last known mailing address or local equivalent methods). These contact attempts will be documented in the participant’s study file.
- Should the participant continue to be unreachable, he or she will be considered to have withdrawn from the study with a primary reason of lost to follow-up.

# 8 STUDY ASSESSMENTS AND PROCEDURES

## 8.1 ENDPOINT AND OTHER NON-SAFETY ASSESSMENTS

| **Table 1. Measures in Laboratory or Home** | | | |
|---|---|---|---|
| **Measure** | **Measurement 1: Baseline**<br><br>**(1-2 h)** | **Measurement 2: Follow-up 1**<br><br>**(4 months)**<br><br>**(1-2 h)** | **Measurement 3: End of study (6 months)**<br><br>**(1-2 h)** |
| **Heart rate and blood pressure** | ✔ | ✔ | ✔ |
| **Anthropometrics (Height, weight, waist, and hip circumference)** | ✔ | ✔ | ✔ |
| **Body composition**<br><br>**(Bioelectrical impedance)** | ✔ | ✔ | ✔ |
| **Fasting blood glucose, and lipid profile** | ✔ | ✔ | ✔ |
| **Cognitive tests** | ✔ | ✔ | ✔ |
| **Physical function tasks** | ✔ | ✔ | ✔ |
| **Muscular and aerobic fitness** | ✔ | ✔ | ✔ |
| **Other** | | | ✔ |
| The following tests and procedures will be conducted in the numbered order. All measurements will be made in a secure location to ensure privacy. | | | |

1. Resting heart rate and blood pressure: Heart rate and blood pressure will be measured using an automatically inflatable blood pressure arm cuff and monitor. These measures will be conducted in triplicate after a rest of 5 minutes between each measure.
2. Anthropometrics: Height and weight will be measured using a stadiometer and a standard digital weight scale, respectively, and circumferences will be measured by a trained research staff member using a measuring tape (in triplicate).
3. Body composition: Whole body percent fat and lean mass will be measured using Bioelectrical Impedance (BIA) and will be conducted by trained research staff. During the test, the participant will step bare feet on an electronic scale and hold handheld conduction electrodes. A weak electric current will flow through the body and the voltage is measured in order to calculate impedance of the body and thereby, the % of body fat and lean mass. The test will be completed within 30 s. The participant will not feel anything, and this test is similar to stepping on a weighing scale.
4. Blood glucose and lipid profiles: We will measure this using finger-stick blood analyses with a CardioChek Plus device (PTS Diagnostics). The CardioChek Plus is a certified fast, accurate and efficient portable analyzer capable of obtaining a full lipid profile and glucose readings in 90 seconds from a finger stick strip blood sample (15 – 40 micro Liters). The participant's blood will not be stored, as the finger stick analyses are immediate, and the measuring strips are discarded appropriately after the test.
5. Cognitive tests: The participant will complete a computer-administered test that measures various aspects of his or her cognitive abilities (executive function, attention skills, episodic memory, working memory and processing speed).
6. Physical function tasks: The participants' pattern of walking (Gait), endurance, strength and balance will be evaluated by a series of performance assessments, which are safe and practical. These assessments mimic daily activities. Details of these performance assessments are as follows:

   (i) Gait: the participant will be asked to walk at his or her usual pace on a 4-meter course twice. The researchers will time each of these walks with a stopwatch.

   (ii) Endurance: the participant will first be asked to perform a series of chair stands. Beginning from a sitting position, the participant will be asked to stand up and then sit down five times in a row, as fast as he or she can, without using his or her arms to help.

   (iii) Strength: the participant will be asked to squeeze a hand-held device to determine the maximum force he or she can produce with each hand repeating the test three times for the left and right hands.

   (iv) Balance: the participant will first be asked to stand in different positions while keeping his or her balance first with eyes open and then with eyes closed. The tester will demonstrate what to do and will be nearby to steady the participant if he or she needs it.

7. Muscular and Aerobic Fitness: We will assess the participant's fitness with a series of short assessments explained below. A trained exercise physiologist will spot and supervise all exercises to ensure safety and proper technique. All testing procedures will be preceded with proper warm-up and instructions to minimize any risks. Details of these assessments are as follows:

   (i) Muscular endurance: We will assess the endurance of the participant's lower and upper body by asking he or she to perform as many repetitions as possible. For the upper body we will do the Standard Push Up test where participants will perform as many pushups as possible. For the lower

body we will use the Repetitive Squat Test where the participant stands with feet 15 cm apart, squats until the thighs are parallel to the floor, and returns to an upright position. We will record the number of repetitions. The participant is free to terminate the tests at any moment either upon voluntary exhaustion or lack of desire to continue.

(ii) Aerobic Fitness- We will assess aerobic fitness using the 6-minute walk test. This is a simple test that requires no specialized equipment and only requires the participant to walk on a flat, hard surface for a period of 6 min. The total distance walked during this duration is measured using a measuring wheel.

8. Other: (i) 7-14 days after baseline, participants in the control group will have a virtual 30-min face-to-face counseling session with the researchers on: i) benefits of reducing sedentary behavior and increasing physical activity, and ii) various strategies to decrease workplace and home sedentary behaviors and initiate a physically active lifestyle.

   (ii) Participants in the treatment group will have a virtual 60-min face-to-face counseling session via a HIPAA complaint ZOOM session. This session will include the same components received by the control group supplemented with an additional 30-min of conversation to further personalize his or her physical activity prescription plan and create goals related to physical activity and sedentary behavior lifestyle choices. During this time the researcher will ask the participant in the treatment group questions about his or her schedule, preferences, health-related behaviors, and other behaviors related to work schedule, hobbies/leisure interests, family dynamics, home and work environment, means of daily transportation, etc. that can be used to help support the participant towards achieving his or her physical activity and sedentary behavior lifestyle goals.

   (iii) After study completion, participants will be asked to complete a follow-up interview in person during the end of study follow-up (month 6). The interview will take no longer than 15-20 minutes. The conversations may be recorded only with the participant's permission. Once the audio recordings are transcribed, the audio recordings will be immediately destroyed.

| **Table 2. Self-completed Measures (1 – 1.5 hours)** | | | |
|---|---|---|---|
| **Measure** | **Baseline** | **Month 4 follow up** | **Month 6 follow up** |
| **General Health and Health Related Quality of Life** | ✔ | | |
| **Perceived Competence Scale for Participating in** | ✔ | ✔ | ✔ |

| | | | |
|---|---|---|---|
| **Regular Physical Activity** | | | |
| **Short Health Care Climate Questionnaire** | | ✔ | |
| **Exercise Self-Regulation Questionnaire** | ✔ | ✔ | ✔ |
| **Basic Need Satisfaction in Relationships Scale** | ✔ | ✔ | ✔ |
| **Diet** | ✔ | ✔ | ✔ |
| **Activity Monitoring** | ✔ | ✔ | ✔ |

After completing the measurements in Table 1, participants will be given questionnaires and small activity monitors. It will take approximately 1-1.5 hours to complete the questionnaires. A research staff member will collect the questionnaires from the participant after a week.

1. <u>Perceived Competence Scale for Participating in Regular Physical Activity</u>: This is a 4-item questionnaire that assesses a participant's feelings of competence about engaging in physical activity.
2. <u>Short Health Care Climate Questionnaire</u>: This questionnaire has 6 items that assess a participant's perceptions of the degree to which the companion is supportive of autonomy; i.e., that the Companion considers the participants perspective and provides appropriate and meaningful feedback and opportunities for choice.
3. <u>Exercise Self-Regulation Questionnaire</u>: This is a 31- item self-report to measure the ability of the participant to regulate behavior to achieve one's goals
4. <u>Basic Need Satisfaction in Relationships Scale</u>: This scale addresses need satisfaction in general in one's life. It has 21 items assessing the three needs for competence, autonomy, and relatedness.
5. <u>Diet</u>: The participant will report frequency and portion size of foods consumed during the past month from a list of 70 commonly consumed food items on a questionnaire.
6. <u>Activity Monitoring</u>: physical activity measurement will be conducted in two ways.
   <u>Firstly</u>, using a thigh worn monitor. This measurement will be conducted for a period of 7-days at baseline and after 4 and 6 months. For this measurement the participant will wear 1 small lightweight (≈ 0.5 ounces) activity monitor (activPAL3™, PAL Technologies, Glasgow, UK; product specs: http://www.palt.com/pals/ )on his or her dominant thigh, which will be adhered to his or her skin using a hypoallergenic medical grade tape (3M Tegaderm ™ Film, 3M, Minneapolis, MN; product specs: *https://www.3m.com/3M/en_US/company-us/all-3m-products/~/3M-Tegaderm-Transparent-*

*Film-Dressing/?N=5002385+3293321979&preselect=3293786499&rt=rud* ). During the 7-day measurement periods, the participant will also complete a record of times the monitors were removed from his or her person. These thigh sensor data will not be transmitted wirelessly. The researchers will download the data from the devices upon collecting them from the participants. The researchers will go to the participant's office/home to collect these.
Secondly, during the entire duration of the study, including the three 7-day physical measurement periods with the thigh activity monitor, the participant will be asked to wear a small wrist sensor (≈ 0.64 ounces) [(i). general exercise group: ActiGraph GT9X Link; (ii). lifestyle physical activity group: CentrePoint Insight Watch, Actigraph Corp. LLC, Pensacola, FL; product specs for GT9X available at https://www.actigraphcorp.com/actigraph-link/ and for Insight Watch available at https://www.actigraphcorp.com/cpiw/). These devices will be worn on the non-dominant wrist on a watchstrap.

Participants in the control group will receive a new GT9X monitor every 1-4 weeks to ensure the devices always have enough battery life to measure physical activity. The researchers will go to the participant's preferred location (office or home) to provide a new sensor. These wrist sensor data will not be transmitted wirelessly. The researchers will download the data from the devices upon collecting them from the participants.

Participants in the treatment group will receive an Insight watch that is capable of passively uploading motion data so that the researchers may get an understanding of his or her daily movement patterns. This information is confidential and will be used for research purposes only. No other data besides data about the participant's movement is gathered using any of the sensors. The researchers will come to the participant's office or home at the beginning of the study to install the data capturing systems (i.e., data transfer hubs). The decision on the location to install the hub (i.e., home vs office) will be determined in consultation with the participant. The location will be where the participant spends the most part of the week during the waking day. These data hubs are small devices that require a power source connection to communicate wirelessly with the device the participant will be wearing and relay data to the researcher. The researchers will uninstall the data hubs following completion of study participation either after the month 6 follow up or prior to that should the participant decide to drop out of the study.

Participants in the control group will also have the battery life of his or her wrist-worn activity monitor frequently monitored by the researchers via the passive data transfer system. When the researchers detect that the device has a low battery (battery life is approximately 2-3 weeks), the participant will be prompted to schedule a time to receive a new device from the researchers. The researcher will go to the participant's office or home to collect the monitor and return it charged by the next day to commence the daily physical activity measurement.

We will encourage participants in both groups to wear the sensors as much as possible so that: 1) we may be able to understand the differences in daily/weekly physical behavior change effects between the two intervention approaches, and 2) we may be able to tailor daily recommendations of participants in the treatment group to maximize and maintain his or her gains and to help meet his or her goals. Participants may remove the sensors for brief intervals throughout the day (e.g., for showering). The researchers may provide participants in the treatment group with retrospective feedback of his or her physical activity measurements via text-message frequently (4 to 7 times a day at intervals of 1 to 4 hours between 7 am – 10 pm), following the virtual session (Table 1 #8). To

reiterate, daily retrospective feedback (health coaching) will last for 4 months, concluding at the month-4 follow up.

Change in outcome measurements will be provided to participants after the follow up at month 6, when requested by the participant.

## 8.2 SAFETY ASSESSMENTS

During the group exercise sessions, participants will be informed that if they feel tired or are uncomfortable to please tell the researchers and the session will be stopped or resumed after a break.

As participants will have been fasting (no food or beverage other than water) for 12 hours prior to outcome measurement, participants will be asked if they are feeling physically alright and will be offered a snack after the finger-stick blood measure. They will be thereafter instructed to resume their regular diet. Participants will also be encouraged to bring their own food if the prefer when testing in the laboratory.

Individuals with skin allergies or skin sensitivities to adhesive tape will be excluded from the study. Tape is required to adhere sensors to the skin to measure the primary outcome.

Should the participant score low on cognitive function test they will asked if they would like to be provided with a resource handbook for dealing with cognitive dysfunction [28].

## 8.3 ADVERSE EVENTS AND SERIOUS ADVERSE EVENTS

### 8.3.1 DEFINITION OF ADVERSE EVENTS

Adverse (AE) and Serious Adverse Events (SAE) will be defined per guidelines from the Office of Human Research Protections (OHRP) of the Department of Health and Human Services.

An adverse event is any untoward medical occurrence in a participant, whether or not it is causally related to the study.

### 8.3.2 DEFINITION OF SERIOUS ADVERSE EVENTS

A serious adverse event is any experience that results in any of the following outcomes: death, is life threatening, inpatient hospitalization or prolongation of hospitalization, a persistent or significant disability/incapacity. Important medical events that may not result in death, be life-threatening, or require hospitalization may be considered a serious adverse event when, based upon appropriate medical judgment, they may jeopardize the patient or participant and may require medical or surgical intervention to prevent one of the outcomes listed in this definition.

### 8.3.3 CLASSIFICATION OF AN ADVERSE EVENT

#### 8.3.3.1 SEVERITY OF EVENT

- Mild: Awareness of a sign or symptom but easily tolerated.
- Moderate: Discomfort sufficient to cause interference with usual activity or to affect clinical status.
- Severe: Incapacitating with inability to do usual activity or to significantly affect clinical status.
- Life Threatening: The participant was at immediate risk of death from the adverse event as it occurred.

#### 8.3.3.2 RELATIONSHIP TO STUDY INTERVENTION/EXPERIMENTAL MANIPULATION

All AEs will have their relationship to study intervention or study participation assessed with a level of specificity appropriate to the study design. The clinician's assessment of an AE's relationship to study intervention (drug, biologic, device, behavioral) is part of the documentation process, but it is not a factor in determining what is or is not recorded in the study. If there is any doubt as to whether a clinical observation is an AE, the event should be recorded. Some protocols may use a binary assessment (related/not related); others may have a scale of relatedness. Evaluation of relatedness must consider etiologies such as natural history of the underlying disease, concurrent illness, concomitant therapy, study-related procedures, accidents, and other external factors. In a clinical trial, the study intervention must always be suspect.

The Investigator will also assess the relationship of any adverse event to study, based upon available information, using the following guidelines:

- 0 = Unlikely: No temporal association, or the cause of the event has been identified.
- 1 = Possible: Temporal association, but other etiologies are likely to be the cause; however, involvement of the study procedures cannot be excluded.
- 2 = Probable: Temporal association, other etiologies are possible, but not likely.

#### 8.3.3.3 EXPECTEDNESS

The safety officer in conjunction with the PI will determine if an AE is expected or unexpected. An AE will be considered unexpected if the nature, severity, or frequency of the event is not consistent with the risk information previously described for the study procedures.

### 8.3.4 TIME PERIOD AND FREQUENCY FOR EVENT ASSESSMENT AND FOLLOW-UP

All adverse events that are both serious and unexpected will be reported by the pilot PI and the Roybal Center PI to the IRB's, NIA PO and to the Safety Officer, within 48 hours of the study's knowledge of SAE. The summary of all other SAEs will be reported to NIA PO and to the Safety Officer, quarterly, unless otherwise requested by the Safety Officer.

### 8.3.5 ADVERSE EVENT REPORTING

Adverse events will be reported by the PI of the Pilot project to the PI of the Roybal Center and the head of the IRB at Northeastern. The Roybal Center PI will report the problems or events to the head of the

IRB at Brandeis University and the NIA Program Officer, and the Safety Officer within 48 h by fax or email according to the Northeastern's IRB written guidelines for interventional studies. All adverse events that are both serious and unexpected (i.e., that have not been previously reported for the study's intervention) will be reported by the pilot PI and the Roybal Center PI to the IRB's, NIA PO and to the Safety Officer, within 48 hours of the study's knowledge of SAE. The summary of all other SAEs will be reported to NIA PO and to the Safety Officer, quarterly, unless otherwise requested by the Safety Officer. All deaths in greater than minimal risk studies require expedited reporting (usually within 24 hours of study's knowledge of death). The report of death will be submitted to NIA Program Officer and to the Safety Officer and the institutional IRB's by the pilot PI and/or the Roybal Center PI.

### 8.3.6 SERIOUS ADVERSE EVENT REPORTING

See section 8.3.5

### 8.3.7 REPORTING EVENTS TO PARTICIPANTS

NA

### 8.3.8 EVENTS OF SPECIAL INTEREST

NA

### 8.3.9 REPORTING OF PREGNANCY

NA

## 8.4 UNANTICIPATED PROBLEMS

### 8.4.1 DEFINITION OF UNANTICIPATED PROBLEMS

Unanticipated problems are those that:

- Are unexpected in terms of nature, severity, or frequency, given (a) the research procedures that are described in the protocol-related documents and (b) the characteristics of the study population;
- Are related or possibly related to participation in the research suggest that the research places participants and others at greater risk of harm than was previously known or recognized

### 8.4.2 UNANTICIPATED PROBLEMS REPORTING

See section 8.3.5.

### 8.4.3 REPORTING UNANTICIPATED PROBLEMS TO PARTICIPANTS

NA

# 9 STATISTICAL CONSIDERATIONS

## 9.1 STATISTICAL HYPOTHESES

- Primary Endpoint(s): Daily total and patterns of physical.

The treatment group using Companion will show greater improvements in daily activity and sedentary behavior outcomes than controls after 16 and 24 weeks. Alternatively, our null hypothesis is that there will be no difference in the treatment group for daily activity and sedentary behavior outcomes after 16 and 24 weeks.

## 9.2 SAMPLE SIZE DETERMINATION

Sample size calculation will be based on conservative power estimates derived using change in daily sedentary behavior among participants (N=12) in our prior intervention [29], which recruited a similar sedentary population (i.e., middle-aged overweight and/or obese seated office workers) and delivered intervention components that encouraged "sitting less and moving more." This study was a behavior change study using treadmill desks, which although not similar to Companion in terms of intervention delivery, had similar characteristics of being "always-available" and "move more sit less" behavior change goals. Study arms with at least 17/group were determined to provide 85% power (5% two-tailed significance) to detect an effect size of 1.06 between groups. A standard deviation of 2.8 h/day for change in total sedentary behavior observed in our prior intervention corresponds to a mean detectable difference of -2.96 sedentary h/day between groups. Our sample size of 23/group accounts for an attrition rate of 26%. Hypothesis testing for all outcomes will entail an intent-to-treat two-tailed design with a type I error of 0.05.

## 9.3 POPULATIONS FOR ANALYSES

Intention-to-Treat (ITT) Analysis Population (i.e., all randomized participants)

## 9.4 STATISTICAL ANALYSES

### 9.4.1 GENERAL APPROACH

Descriptive statistics, for all normally distributed continuous data will be presented as means with standard deviations. Hypothesis testing will entail an intent-to-treat two-tailed design with a nominal

type I error of 0.05. All participants will be included in the intent-to-treat analyses according to randomization regardless of their subsequent status. Missing data due to losses to follow-up will be assumed to be at random. Random intercept mixed linear models that account for repeated measures will be used to compare primary and exploratory outcomes measured at various timepoints during the study. Models will be adjusted for covariates and the baseline value of the outcome variable where necessary.

### 9.4.2 ANALYSIS OF THE PRIMARY ENDPOINT(S)

The primary endpoint will be summarized from body-worn sensor data and is a continuous variable. Hypothesis testing entailed an intent-to-treat two-tailed design with a nominal type I error of 0.05. Random intercept mixed linear models that account for repeated measures will be used to analyze the primary outcome. Models will be adjusted for covariates and the baseline value of the outcome variable where necessary. Post-hoc pairwise comparisons for primary outcomes will be adjusted for multiple testing using Bonferroni corrections. Results will be presented as Least-squares means (LSMEANS) with 95% confidence intervals. Effect sizes and confidence intervals will be calculated for all comparisons.[23,24] Additionally, we will conduct sensitivity analyses where missing outcome data attributable to random factors will be imputed using joint multiple imputation and analyzed using mixed linear modelling adjusted for covariates.[25,26]

### 9.4.3 ANALYSIS OF THE SECONDARY ENDPOINT(S)

NA

### 9.4.4 SAFETY ANALYSES

NA

### 9.4.5 BASELINE DESCRIPTIVE STATISTICS

The intervention and control group will be compared for differences in baseline characteristics using linear mixed models.

### 9.4.6 PLANNED INTERIM ANALYSES

NA

### 9.4.7 SUB-GROUP ANALYSES

NA

### 9.4.8 TABULATION OF INDIVIDUAL PARTICIPANT DATA

Individual participant data will be listed by measure and time point in a database.

#### 9.4.9 EXPLORATORY ANALYSES

Random intercept mixed linear models that account for repeated measures will be used to analyze exploratory outcomes. Results will be presented as Least-squares means (LSMEANS) with 95% confidence intervals. Effect sizes and confidence intervals will be calculated for all comparisons.[23,24]Analyses for exploratory outcomes will be considered to be hypothesis-generating and thus, will not be adjusted for multiple testing (i.e., α= 0.05). Effect sizes and confidence intervals will be calculated for all comparisons.[23,24]

# 10 SUPPORTING DOCUMENTATION AND OPERATIONAL CONSIDERATIONS

## 10.1 REGULATORY, ETHICAL, AND STUDY OVERSIGHT CONSIDERATIONS

### 10.1.1 INFORMED CONSENT PROCESS.

#### 10.1.1.1 CONSENT/ASSENT AND OTHER INFORMATIONAL DOCUMENTS PROVIDED TO PARTICIPANTS

Consent forms describing in detail the study intervention, study procedures, and risks will be given to the participant and written documentation of informed consent will be completed prior to starting the study intervention. The following consent materials are submitted with this protocol:

- Informed consent document
- Recruitment Flyer
- Recruitment Email template
- Recruitment quota reached email template

#### 10.1.1.2 CONSENT PROCEDURES AND DOCUMENTATION

The project manager and/or graduate student trained in the consent process will obtain informed consent and potential participants will receive an oral explanation regarding study procedures and associated risks and benefits. Participants will be made aware that their participation is voluntary, and they have the right to discontinue participation at any time and for any reason. Risks associated with the study will be clearly outlined in the informed consent document. Participants will have time to read the consent form and the experimenter will address participants' questions and concerns prior to signing the consent form. The participant will be explicitly told that they can take as long as needed to read over and ask questions. We will ensure confidentiality during the consent process and allow participants sufficient time to decide on participation. Signed consent will be obtained prior to admission to the study. This will include a complete description of the study and will include telephone numbers to contact the principal investigator and Institutional Review Board (IRB) if they have questions.

### 10.1.2 STUDY DISCONTINUATION AND CLOSURE

This study may be temporarily suspended or prematurely terminated if there is sufficient reasonable cause. Written notification, documenting the reason for study suspension or termination, will be provided by the suspending or terminating party to study participants, investigator, funding agency, and regulatory authorities. If the study is prematurely terminated or suspended, the Principal Investigator (PI) will promptly inform study participants, the Institutional Review Board (IRB), and sponsor/funding agency and will provide the reason(s) for the termination or suspension. Study participants will be contacted, as applicable, and be informed of changes to study measurement schedule.

Circumstances that may warrant termination or suspension include, but are not limited to:

- Determination of unexpected, significant, or unacceptable risk to participants
- Demonstration of efficacy that would warrant stopping
- Insufficient compliance of study staff to the protocol (i.e., significant protocol violations)
- Data that are not sufficiently complete and/or evaluable
- Determination that the primary endpoint has been met
- Determination of futility

The study may resume once concerns about safety, protocol compliance, and data quality are addressed, and satisfy the funding agency, sponsor, IRB, or other relevant regulatory or oversight bodies (e.g., Safety Officer).

### 10.1.3 CONFIDENTIALITY AND PRIVACY

Participant confidentiality and privacy is strictly held in trust by the participating investigators, their staff, the safety and oversight monitor(s), and the sponsor(s) and funding agency. This confidentiality is extended to the data being collected as part of this study. Data that could be used to identify a specific study participant will be held in strict confidence within the research team. No personally identifiable information from the study will be released to any unauthorized third party without prior written approval of the sponsor/funding agency.

All research activities will be conducted in as private a setting as possible.

The study safety officer, other authorized representatives of the sponsor or funding agency, and representatives of IRB may inspect all documents and records required to be maintained by the investigator for the participants in this study. The study site will permit access to such records.

The study participant's contact information will be securely stored at the study site for internal use during the study. At the end of the study, all records will continue to be kept in a secure location for as long a period as dictated by the reviewing IRB, Institutional policies, or sponsor/funding agency requirements.

Study participant research data, which is for purposes of statistical analysis and scientific reporting, will be stored securely on a password protected computer at Northeastern University. This will not include

the participant's contact or identifying information. Rather, individual participants and their research data will be identified by a unique study identification number. At the end of the study, all study databases will be de-identified and archived.

Measures Taken to Ensure Confidentiality of Data Shared per the NIH Data Sharing Policies
It is NIH policy that the results and accomplishments of the activities that it funds should be made available to the public (see https://grants.nih.gov/policy/sharing.htm). The PI will ensure all mechanisms used to share data will include proper plans and safeguards for the protection of privacy, confidentiality, and security for data dissemination and reuse (e.g., all data will be thoroughly de-identified and will not be traceable to a specific study participant). Plans for archiving and long-term preservation of the data will be implemented, as appropriate.

### 10.1.4 FUTURE USE OF STORED SPECIMENS AND DATA

NA

### 10.1.5 KEY ROLES AND STUDY GOVERNANCE

| **Principal Investigator** | **Independent Safety Monitor** |
|---|---|
| Dinesh John, Associate Professor | Paul Thompson, MD |
| Northeastern University | Hartford Hospital |
| 360 Huntington Avenue, Boston, MA-02115 | 85 Jefferson Street, Suite 704<br>Hartford, CT 06106 |
| 6173735695 | 860-972-1793 |
| d.john@northeastern.edu | paul.thompson@hhchealth.org |

### 10.1.6 SAFETY OVERSIGHT

Safety oversight will be guided by a Data and Safety Monitoring Plan under the direction of a Safety Monitor. The Safety Monitor will be independent from the study conduct and free of conflict of interest. The Safety Officer will meet biannually with the PI to review standardized reports. The Safety Officer will review the progress of recruitment and retention of participants, compliance with the protocol, and operating procedures. If he/she identifies any safety issue, they may request additional data and propose

specific analyses and make recommendations to the PI regarding recruitment, retention, compliance, and safety issues

#### 10.1.7 CLINICAL MONITORING

NA

#### 10.1.8 QUALITY ASSURANCE AND QUALITY CONTROL

Quality control (QC) procedures will be implemented as follows:

**Informed consent ---** Study staff will review both the documentation of the consenting process as well as the completed consent documents. This review will evaluate compliance with Good Clinical Practice, accuracy, and completeness. Feedback will be provided to the study team to ensure proper consenting procedures are followed.

**Source documents and the electronic data ---** Data initially captured on source documents (see **Section 10.1.9, Data Handling and Record Keeping**) will ultimately be entered into the study database. To ensure accuracy site staff will compare a representative sample of source data against the database, targeting key data points in that review.

**Intervention Fidelity** — Consistent delivery of the study interventions will be monitored throughout the intervention phase of the study. Procedures for ensuring fidelity of intervention delivery are described in **Section 6.2.1, Interventionist Training and Tracking**.

**Protocol Deviations** – The study team will review protocol deviations on an ongoing basis and will implement corrective actions when the quantity or nature of deviations are deemed to be at a level of concern.

Should independent monitoring become necessary, the PI will provide direct access to all trial related sites, source data/documents, and reports for the purpose of monitoring and auditing by the sponsor/funding agency, and inspection by local and regulatory authorities.

#### 10.1.9 DATA HANDLING AND RECORD KEEPING

##### 10.1.9.1 DATA COLLECTION AND MANAGEMENT RESPONSIBILITIES

Data collection will be the responsibility of the clinical trial staff at the site under the supervision of the site investigator. The investigator will be responsible for ensuring the accuracy, completeness, legibility, and timeliness of the data reported.

All source documents will be completed in a neat, legible manner to ensure accurate interpretation of data.

Hardcopies of outcome measurement worksheets will be provided for use as source document worksheets for recording data for each participant consented/enrolled in the study. Data recorded on an electronic form derived from source documents will be consistent with the data recorded on the source documents.

Hard copies of source data will be de-identified and stored in locked cabinet inside a locked office in the Human Performance and Exercise Science Lab. Only authorized research personnel will have access to this office. Signed consent forms will also be stored in this same locked cabinet

Should the participant agree to having his or her study exit interview audio recorded, the recording will be destroyed immediately after the interview has been transcribed.

Physical activity data collected from wearable sensors in the treatment group will be uploaded to a secure cloud-based storage system (CentrePoint, ActiGraph LLC, Pensacola, FL), which will allow authorized study personnel to securely and remotely access the data. The CentrePoint system leverages existing cloud-based technologies to provide a robust, scalable solution that implements industry best practices. CentrePoint utilizes Microsoft Azure's cloud computing platform for hosting sites/services, data processing, subject/study management, and data storage, and uses AWS Relational Database Service (RDS) for data analytics.

Using WhatsApp for daily text-message based communication will ensure that the participants' conversations with the researchers remain secured and only visible to them and the researchers. This is made possible by WhatsApp's end-to-end encryption, which is automatically available to all users of the app. Many messaging apps only encrypt messages between the sender and the receiver, but WhatsApp's end-to-end encryption ensures that only the sender and the person he or she is communicating with, can read what is sent, and nobody in between, not even employees of WhatsApp. This is because messages are secured with a lock, and only the recipient and the sender have the special key needed to unlock and read them. For added protection, every message sent on WhatsApp has its own unique lock and key. All of this happens automatically.

#### 10.1.9.2 STUDY RECORDS RETENTION

Study documents will be retained until at least 2 years have elapsed since the formal discontinuation of clinical development of the study intervention or as per IRB guidelines.

### 10.1.10 PROTOCOL DEVIATIONS

A protocol deviation is defined as any noncompliance with the clinical trial protocol. The noncompliance may be either on the part of the participant, the investigator, or the study site staff. As a result of deviations, corrective actions will be developed by the site and implemented promptly.

These practices are consistent with ICH GCP:

- Section 4.5 Compliance with Protocol, subsections 4.5.1, 4.5.2, and 4.5.3
- Section 5.1 Quality Assurance and Quality Control, subsection 5.1.1
- Section 5.20 Noncompliance, subsections 5.20.1, and 5.20.2.

It will be the responsibility of the site investigator to use continuous vigilance to identify and report deviations within 7 working days of identification of the protocol deviation. All deviations will be addressed in study source documents, reported to the funding agency. Protocol deviations will be sent to the reviewing Institutional Review Board (IRB) per their policies. The site investigator will be responsible for knowing and adhering to the reviewing IRB requirements.

#### 10.1.11 PUBLICATION AND DATA SHARING POLICY

This study will be conducted in accordance with the following publication and data sharing policies and regulations:

National Institutes of Health (NIH) Public Access Policy, which ensures that the public has access to the published results of NIH funded research. It requires scientists to submit final peer-reviewed journal manuscripts that arise from NIH funds to the digital archive PubMed Central upon acceptance for publication.

This study will comply with the NIH Data Sharing Policy and Policy on the Dissemination of NIH-Funded Clinical Trial Information and the Clinical Trials Registration and Results Information Submission rule. As such, this trial will be registered at ClinicalTrials.gov, and results information from this trial will be submitted to ClinicalTrials.gov. In addition, every attempt will be made to publish results in peer-reviewed journals. Data from this study may be requested from other researchers 4 years after the completion of the primary endpoint by contacting the PI. Considerations for ensuring confidentiality of these shared data are described in Section 10.1.3.

#### 10.1.12 CONFLICT OF INTEREST POLICY

Any conflict of interest of persons who have a role in the design, conduct, analysis, publication, or any aspect of this trial will be disclosed and managed. Furthermore, persons who have a perceived conflict of interest will be required to have such conflicts managed in a way that is appropriate to their participation in the design and conduct of this trial.

### 10.2 ADDITIONAL CONSIDERATIONS

NA

## 10.3 ABBREVIATIONS AND SPECIAL TERMS

The list below includes abbreviations utilized in this template. However, this list should be customized for each protocol (i.e., abbreviations not used should be removed and new abbreviations used should be added to this list). Special terms are those terms used in a specific way in the protocol. For instance, if the protocol has therapist-participants and patient-participants, those terms could be included here for purposes of consistency and specificity.

| | |
|---|---|
| AE | Adverse Event |
| CFR | Code of Federal Regulations |
| GCP | Good Clinical Practice |
| ICH | International Council on Harmonisation |
| IRB | Institutional Review Board |
| LSMEANS | Least-squares Means |
| NIH | National Institutes of Health |
| NIH IC | NIH Institute or Center |
| OHRP | Office for Human Research Protections |
| PI | Principal Investigator |
| QC | Quality Control |
| SAE | Serious Adverse Event |
| UP | Unanticipated Problem |
| US | United States |
| CPR | Cardio-pulmonary resucitation |

## 10.4 PROTOCOL AMENDMENT HISTORY

The table below is intended to capture changes of IRB-approved versions of the protocol, including a description of the change and rationale. A **Summary of Changes** table for the current amendment is located in the **Protocol Title Page**.

| Version | Date | Description of Change | Brief Rationale |
|---|---|---|---|
| | | | |
| | | | |
| | | | |
| | | | |
| | | | |
| | | | |
| | | | |
| | | | |
| | | | |
| | | | |
| | | | |
| | | | |
| | | | |
| | | | |

| | | | |
|---|---|---|---|
| | | | |
| | | | |
| | | | |
| | | | |
| | | | |
| | | | |
| | | | |
| | | | |
| | | | |

# 11 REFERENCES

1. Castaneda C, Layne JE, Munoz-Orians L, et al. A randomized controlled trial of resistance exercise training to improve glycemic control in older adults with type 2 diabetes. Diabetes care. 2002;25(12):2335-2341.
2. Nelson M, Layne J, Bernstein M, Nuernberger A. Castaneda C,* Kaliton D, Hausdorff J, Judge J, Buchner DM, Roubenoff R, Fiatarone Singh MA. The effects of multidimensional home based exercise on functional performance in the elderly. Journal of Gerontology A. 2004;59:154-160.
3. Devereux-Fitzgerald A, Powell R, Dewhurst A, French DP. The acceptability of physical activity interventions to older adults: A systematic review and meta-synthesis. Social Science & Medicine. 2016;158:14-23.
4. Erickson KI, Grove GA, Burns JM, et al. Investigating gains in neurocognition in an Intervention Trial of Exercise (IGNITE): Protocol. Contemporary clinical trials. 2019;85:105832.
5. Exercise Programs That Promote Senior Fitness. National Council on Aging. ncoa.org/center-for-healthy-aging/basics-of-evidence-based-programs/physical-activity-programs-for-older-adults/. Accessed2019.
6. Pahor M, Guralnik JM, Ambrosius WT, et al. Effect of structured physical activity on prevention of major mobility disability in older adults: the LIFE study randomized clinical trial. JAMA : the journal of the American Medical Association. 2014;311(23):2387-2396.
7. Pavey T, Taylor A, Hillsdon M, et al. Levels and predictors of exercise referral scheme uptake and adherence: a systematic review. J Epidemiol Community Health. 2012;66(8):737-744.
8. Picorelli AM, Pereira LS, Pereira DS, Felicio D, Sherrington C. Adherence to exercise programs for older people is influenced by program characteristics and personal factors: a systematic review. J Physiother. 2014;60(3):151-156.
9. Shubert TE. Evidence-based exercise prescription for balance and falls prevention: a current review of the literature. J Geriatr Phys Ther. 2011;34(3):100-108.
10. Williams NH, Hendry M, France B, Lewis R, Wilkinson C. Effectiveness of exercise-referral schemes to promote physical activity in adults: systematic review. Br J Gen Pract. 2007;57(545):979-986.
11. Courneya KS, Karvinen KH, McNeely ML, et al. Predictors of adherence to supervised and unsupervised exercise in the Alberta Physical Activity and Breast Cancer Prevention Trial. Journal of physical activity & health. 2012;9(6):857-866.
12. Lacroix A, Kressig RW, Muehlbauer T, et al. Effects of a Supervised versus an Unsupervised Combined Balance and Strength Training Program on Balance and Muscle Power in Healthy Older Adults: A Randomized Controlled Trial. Gerontology. 2016;62(3):275-288.
13. Meijer EP, Westerterp KR, Verstappen FT. Effect of exercise training on total daily physical activity in elderly humans. European journal of applied physiology and occupational physiology. 1999;80(1):16-21.
14. Meijer EP, Westerterp KR, Verstappen FT. Effect of exercise training on physical activity and substrate utilization in the elderly. International journal of sports medicine. 2000;21(7):499-504.
15. Morio B, Montaurier C, Pickering G, et al. Effects of 14 weeks of progressive endurance training on energy expenditure in elderly people. The British journal of nutrition. 1998;80(6):511-519.
16. Drenowatz C. Reciprocal Compensation to Changes in Dietary Intake and Energy Expenditure within the Concept of Energy Balance. Adv Nutr. 2015;6(5):592-599.
17. Melanson EL, Keadle SK, Donnelly JE, Braun B, King NA. Resistance to exercise-induced weight loss: compensatory behavioral adaptations. Medicine and science in sports and exercise. 2013;45(8):1600-1609.

18. Doucet E, McInis K, Mahmoodianfard S. Compensation in response to energy deficits induced by exercise or diet. Obesity reviews : an official journal of the International Association for the Study of Obesity. 2018;19 Suppl 1:36-46.
19. Melanson EL. The effect of exercise on non-exercise physical activity and sedentary behavior in adults. Obesity reviews : an official journal of the International Association for the Study of Obesity. 2017;18 Suppl 1:40-49.
20. Bankoski A, Harris TB, McClain JJ, et al. Sedentary activity associated with metabolic syndrome independent of physical activity. Diabetes care. 2011;34(2):497-503.
21. Elosua R, Bartali B, Ordovas JM, et al. Association between physical activity, physical performance, and inflammatory biomarkers in an elderly population: the InCHIANTI study. The journals of gerontology Series A, Biological sciences and medical sciences. 2005;60(6):760-767.
22. Physical Activity Guidelines for Americans. Health and Human Services. https://www.hhs.gov/fitness/be-active/physical-activity-guidelines-for-americans/index.html. Published 2018. Accessed 2019.
23. Czaja SJ. Can technology empower older adults to manage their health? Generations. 2015;39(1):46-51.
24. Roess A. The Promise, Growth, and Reality of Mobile Health - Another Data-free Zone. The New England journal of medicine. 2017;377(21):2010-2011.
25. Arguello D, Ponnada A, Cloutier G, Intille S, John D. Communication with a Smartphone-based Virtual Health Companion: A Pilot Study. 2016. (Unpublished work).
26. Katz P, Julian L, Tonner MC, et al. Physical activity, obesity, and cognitive impairment among women with systemic lupus erythematosus. Arthritis Care Res (Hoboken). 2012;64(4):502-510.
27. Napoli N, Shah K, Waters DL, Sinacore DR, Qualls C, Villareal DT. Effect of weight loss, exercise, or both on cognition and quality of life in obese older adults. The American journal of clinical nutrition. 2014;100(1):189-198.
28. Medalia A, Revheim N. Dealing with cognitive dysfunction associaed with psychiatric disabilities: A handbook for families and friends of individuals with psychiatric disorders. New York State Office of Mental Health Family Liaison Bureau. *https://omh.ny.gov/omhweb/cogdys_manual/cogdyshndbk.pdf*. Published 2002. Accessed 2021.
29. John D, Thompson DL, Raynor H, Bielak K, Rider B, Bassett DR. Treadmill workstations: a worksite physical activity intervention in overweight and obese office workers. *J Phys Act Health*. 2011, 8(8), 1034-43.
