# Supplementary material for: Companion: A Pilot Randomized Clinical Trial to Test an Integrated Two-Way Communication and Near-Real-Time Sensing System for Detecting and Modifying Daily Inactivity among Adults >60 Years—Design and Protocol
Source: Sensors (Basel). 2023 Feb 16;23(4):2221. doi: 10.3390/s23042221 (PMC9965440; doi:10.3390/s23042221)
Supplement: Supplementary file 1 [file sensors-23-02221-s001.zip › Supplemental Table S1.pdf]

Supplemental Table S1. Categorization of Companion and User Messages by Objective and Tone

Categorization	Definition
Message Objective	
Educational	A message meant to inform or educate on related health or study information.
Activity related	A message meant to elicit movement/activity.
Commitment reminder	A message meant to motivate intervention adherence by reminding a participant of their goals.
Goal setting	Sets an achievable physical activity goal for the day; delivered early in the day.
Self-monitoring	Informs participant about their progress and aims to motivate them to achieve their physical activity goals.
Goal achievement	Informs the participants whether they achieved their goal for the day and aimed to motivate them to complete future goals; delivered at the end of the day.
Actionable tip	A message that aims to provide strategies on how to better ensure goal achievement.
Positive reinforcement	A message that provides positive feedback when a participant is on track to achieve a pre-set goal or has already attained his or her goal.
Booster message	A message to motivate individuals when they were not tracking to meet or surpass their daily goals.
Personal health	A message that asks about or acknowledges the health of the specific participant (sleep, diet, activity level, etc.).
Inquiry/elicit response	A message that asks a question or encourages a reply from the participant.
Repeated	A message that is sent multiple times throughout a day or duration of the study.
Social	A message meant to develop a relationship or humanize the messenger.
Greeting message	A generic greeting message that precedes an intervention message.
Miscellaneous	A message that does not fall into any of the above categories.

Companion Message Tone	
Informative/educational	A message that aims to educate or inform, often health or study related.
Inquisitive	A message that asks a question.
Supportive/empowering	A message that aims to motivate self-monitoring and intervention adherence.
Threatening/consequential	A message that aims to motivate adherence through acknowledging negative outcomes of failure in maintaining a goal, often educational or statistical.
Humorous	A message that uses humor to encourage adherence or engagement.
Celebratory	A message that celebrates or congratulates a user for accomplishing a goal.
Sympathetic/understanding	A message that acknowledges a failure to achieve a goal and aims to motivate for future goal achievements.
Social	A message meant to develop a relationship or humanize the messenger.
Miscellaneous	A message that does not fall into any of the above categories.
User Message Tone	
Informative	User is answering a question or relaying information about themselves to Companion.
Inquisitive	User asks a question.
Appreciative	User sends a message of gratitude towards a previous message or towards Companion in general.
Negative	User responds negatively or replies "no" to a message eliciting a response.
Humorous	User responds with humor or a message that implies laughter.
Social	User engages with a message or messages through continuing conversation with more than one reply or longer message responses with the aim of developing a relationship with the Companion.
Miscellaneous	Any message that does not easily fit into any of the above categories.
